# Piezo1-directed neutrophil extracellular traps regulate macrophage differentiation during influenza virus infection

**DOI:** 10.1038/s41419-025-07395-5

**Published:** 2025-01-31

**Authors:** Yuexin Wang, Qiuli Yang, Yingjie Dong, Likun Wang, Zhiyuan Zhang, Ruiying Niu, Yufei Wang, Yujing Bi, Guangwei Liu

**Affiliations:** 1 Key Laboratory of Cell Proliferation and Regulation Biology, Ministry of Education, College of Life Sciences, Beijing Normal University, 100875 Beijing, China; 2 State Key Laboratory of Pathogen and Biosecurity, Academy of Military Medical Science, 100080 Beijing, China

**Keywords:** Immunological disorders, Inflammatory diseases

## Abstract

Neutrophils and macrophages are critical for antiviral immunity, but their reciprocal regulatory roles and mechanisms in the response to viral infection remain unclear. Herein, we found that the ion channel Piezo1 directs neutrophil extracellular trap (NET) formation and regulates macrophage functional differentiation in anti-influenza virus immunity. Genetic deletion of Piezo1 in neutrophils inhibited the generation of NETs and M1 macrophage differentiation while driving the development of M2 macrophages during viral infection. Piezo1-directed neutrophil NET DNA directly regulates macrophage differentiation in vitro and in vivo. Mechanistically, neutrophil Piezo1 deficiency inhibited NET DNA production, leading to decreased TLR9 and cGAS-STING signalling activity while inducing reciprocal differentiation from M1 to M2 macrophages. In addition, Piezo1 integrates magnesium signalling and the SIRT2-hypoxia-inducible factor-1 alpha (HIF1α)-dependent pathway to orchestrate reciprocal M1 and M2 macrophage lineage commitment through neutrophil-derived NET DNA. Our studies provide critical insight into the role of neutrophil-based mechanical regulation of immunopathology in directing macrophage lineage commitment during the response to influenza virus infection.

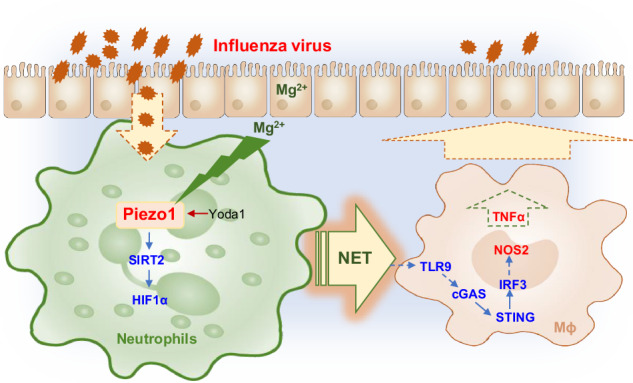

## Introduction

Neutrophils and macrophages are most infiltrated immune cells, which have essential roles at the forefront of defence against pathogen microbial infection [1]. Neutrophils are often the first to be recruited to the pathogen invasion site to phagocytose and present microbial antigens and provide further information for subsequent immune responses, including the recruitment of more macrophages and other immune cells or the regulation of immune cell differentiation [2, 3]. They can phagocytose, degranulate and produce reactive oxygen species (ROS) to play a role in combating pathogens during infection [4, 5]. Recently, neutrophil extracellular traps (NETs) have attracted increased interest from researchers and they have shown that NETs are crucial for the function of neutrophils. NETs are composed of dsDNA and histone and nonhistone proteins, including neutrophil elastase and myeloperoxidase, which regulate NET formation and antimicrobial function [6, 7]. ROS directly activate the protein arginine deaminase 4 (PAD4) to deconcentrate chromatin, which is critically involved in regulating NET formation by neutrophils [3, 8]. In addition, macrophages often regulate immune function by differentiating into type M1 macrophages, increasing the secretion of the proinflammatory cytokine TNFα, reducing the expression of the anti-inflammatory cytokine IL-10 and inducible nitric oxide synthase (NOS2), or differentiating into type M2 macrophages, inhibiting the secretion of proinflammatory cytokines, enhancing the production of anti-inflammatory cytokines and promoting the expression of CD206 [9, 10]. DNA recognition receptor toll-like receptor 9 (TLR9) and cytoplasmic DNA sensor cyclic GMP-AMP synthetase (cGAS) signalling are critically involved in regulating the functional activities of macrophages in infectious diseases [11–14]. However, how neutrophils regulate the function of macrophages in fighting against influenza virus infection remains unclear.

Piezo1 was originally identified as a mechanically activated nonselective cation ion channel with significant permeability to calcium and magnesium ions, is evolutionarily conserved and is involved in the proliferation and development of multiple types of cells in the context of diverse types of mechanical and/or innate stimuli [15–17]. Our study and other previous studies have shown that Piezo1 is critically involved in regulating various types of immune cell functions, including those of dendritic cells (DCs), neutrophils and T cells, in inflammation and cancer [18–21]. However, the regulatory effect of Piezo1 on neutrophils, especially during influenza virus infection, is still unclear.

Here, we found that the response of the neutrophil ion channel sensor Piezo1 influences virus and magnesium signals and directs the reciprocal differentiation of M1 and M2 macrophages. Piezo1 largely acts by integrating the SIRT2–hypoxia–inducible factor-1 alpha (HIF1α)-dependent signalling pathway and regulates the production of NET DNA by neutrophils as well as the signalling activation of TLR9 and cGAS–STING by responding macrophages.

## Materials and methods

### Mice

The animals were maintained in pathogen-free conditions. C57BL/6 *Piezo1*^flox/flox^, *Lyz-Cre*, *Sirt2*^flox/flox^ and *Hif1α*^flox/flox^ mice were obtained from the Jackson Laboratory (Bar Harbor, ME, USA). C57BL/6 mice were obtained from Beijing University Experimental Animal Center (Beijing, China). All of the mice were backcrossed to the C57BL/6 background for at least eight generations and were used at an age of 6–12 weeks. WT control mice were of the same genetic background and, where relevant, included Cre^+^ mice to account for the effects of Cre (no adverse effects due to Cre expression itself were observed in vitro or in vivo).

### Viral infection model

To establish a mouse influenza virus infection model, 50 µl of a mouse-adapted influenza virus strain A/Puerto Rico/8/34 (PR8, H1N1) at a dose of 450 TCID_50_ (half maximal tissue culture infectious dose) was used to infect each mouse intranasally. The mice were killed at 48 h after virus infection, and the bronchoalveolar lavage fluid (BALF) and lungs were harvested as previously described [22]. The left lobe of the lung was used for histological assessment, and the right lobe was used for the analysis of mRNA, protein, flow cytometry, and virus infectious titre. For the depletion of macrophages in vivo, 100 µl of clodronate (clodronate liposomes, Netherlands) or control (PBS) liposomes were injected i.p. daily, as described previously [23]. A magnesium-restricted diet and a matching control diet, which was based on the purified ingredient rodent diet *AIN-76A*, were purchased from Research Diets Inc. (USA).

### Virus titre

The virus infectious titre was measured as previously described [24]. In brief, the lung lobe was harvested at the indicated time points after influenza infection. Lung homogenates were serially diluted with Minimum Essential Medium Eagle (Sigma‒Aldrich) and added to confluent Madin–Darby canine kidney (MDCK) cells (ATCC) in 96-well plates. After 4 days of incubation at 37 °C, virus infectious titres were measured via a TCID_50_ assay on the basis of cytopathic effects.

### Histopathology

Formalin-fixed lungs were processed and embedded in paraffin, sectioned at 5 µm, mounted on positively charged glass slides (Thermo Fisher Scientific), and dried at 60 °C for 30 min, as described previously [25]. Sections were stained with haematoxylin and eosin (H&E). The slides were scanned with a Pannoramic Digital Slide Scanner (SDHISTECH, Budapest, Hungary), and the images were cropped from virtual slides in Pannoramic Viewer as previously described [26]. Lung inflammation severity was graded on the basis of the histological pathological criteria, as described previously [27].

### Immunohistochemistry (IHC)

Lungs from the model mice were collected, fixed in 10% formalin overnight and embedded in paraffin. Formalin-fixed paraffin-embedded tissue was cut into 4 µm sections. The sections were processed using standard protocols for xylene and an alcohol gradient for deparaffinization. After antigen retrieval and unmarking procedures, the sections were incubated with primary antibody anti-Ly6G (Biolegend, San Diego, CA, USA) and anti-F4/80 (Biolegend, San Diego, CA, USA). The sections were incubated with mouse anti-rodent HRP-polymer (Biocare Medical, Concord, USA) for 40 min and then developed with an Ultravision DAB Plus Substrate Detection System (TA-125-QHDX, Thermo Fischer Scientific, Waltham, MA, USA) for 2–5 min at room temperature, followed by haematoxylin staining, dehydration and coverslipping with Permount. Immunohistochemistry (IHC) slides were scanned with a Pannoramic Digital Slide Scanner (SDHISTECH, Budapest, Hungary), and images were cropped from virtual slides in Pannoramic Viewer as previously described [26].

### Neutrophil isolation and cell culture

At 48 h after virus infection, the mouse BALF and lungs were harvested as previously described [22]. The lung tissue was cut into pieces and washed with RPMI 1640. Then, the lung fragments were resuspended in a 2 ml solution of 1 mg/ml collagenase XI (Sigma–Aldrich, St. Louis, MO, USA) and incubated at 37 °C for 30 min. PBS with 1% FBS and 5 mM ethylenediaminetetraacetic acid (EDTA) was used to neutralize the digestion. The cells were washed twice (452 × g, 5 min), resuspended in RPMI 1640, and filtered to remove clumps. CD11b^+^Ly6G^+^ neutrophils were isolated from single-cell suspensions of lungs via cell sorting on a FACSAria (BD Biosciences, Franklin Lake, NJ, USA), as previously described [28, 29].

Human CD34^+^ haematopoietic stem cell (HSC; 2M-101C, Lonza)-derived neutrophils were generated as previously described [25]. In brief, HSCs were cultured in complete DMEM supplemented with 2 mM l-glutamine, 10 mM HEPES, 20 mM 2-ME, 150 U/ml streptomycin, 200 U/ml penicillin, and 10% FBS and stimulated with G-CSF (10 ng/ml, Peprotech, Rocky Hill, NJ, USA). The cultures were maintained at 37 °C in a 5% CO_2_-humidified atmosphere for 7 days. Human peripheral blood CD14^+^ monocyte (2W-400A, Lonza)-derived macrophages were generated as previously described [30]. In brief, monocytes were cultured in complete DMEM supplemented with 2 mM l-glutamine, 10 mM HEPES, 20 mM 2-ME, 150 U/ml streptomycin, 200 U/ml penicillin, and 10% FBS and stimulated with M-CSF (10 ng/ml, Peprotech, Rocky Hill, NJ, USA). The cultures were maintained at 37 °C in a 5% CO_2_-humidified atmosphere for 7 days.

### NET formation assay

Neutrophils were sorted from single-cell suspensions of BALF or spleens from mice. A total of 2 ×10^5^ cells was plated in 200 µl in a 96-well flat bottom plate and incubated for the indicated time. The obtained pure neutrophils were fixed with 4% paraformaldehyde for 10 min and permeabilized with 5% Triton X-100 for 20 min in PBS at room temperature. Neutrophils were incubated with anti-histone H3 antibody (ab5103) for 20 min and detected using Alexa Fluor® 488-conjugated anti-rabbit IgG (H + L) (Cell Signalling Technology). NETs and nucleic acids were detected with SYTOX orange (Thermo Fisher) and Hoechst 33342 (Beyotime), respectively. Z-Stacks (10–30 µm 40X magnification) were taken using an LSM800 instrument equipped with a 488 diode and a Plan-Apochromat 1,3 N/An Oil DIC III objective. For NET area quantification, FIJI software and the particle analysis plugin were used. Only structures depicting NET morphology and positive for SYTOX green were selected for area quantification, and intact granulocyte nuclei were excluded from the analysis. Triplicate wells of each condition were included, as described previously [31].

### NET DNA purification

Sorted splenic neutrophils (2 ×10^7^) from the mice were stimulated with LPS for 4 h. After removal of the supernatant, NETs that had adhered to the bottom were removed by pipetting 2 ml of cold PBS and were subsequently centrifuged at 1000 × *g* at 4 °C for 10 min. The cell-free supernatant containing NETs (DNA‒protein complexes) was collected. The DNA concentration of NETs was measured by spectrophotometry, and the NETs were used for further experiments as described with slight modifications [32].

### Cell cultures and flow cytometry

Spleens were digested with collagenase D, and neutrophils (Ly6G^+^TCR^-^CD19^-^NK1.1^-^F4/80^-^CD11c^-^) were sorted with a FACSAria II (Becton Dickinson, San Diego, CA, USA). BM-derived macrophage generation was performed as previously described [30]. In brief, BM cells were cultured in complete DMEM supplemented with 2 mM L-glutamine, 10 mM HEPES, 20 mM 2-ME, 150 U/ml streptomycin, 200 U/ml penicillin, and 10% FBS and stimulated with M-CSF (10 ng/ml, Peprotech, Rocky Hill, NJ, USA). The cultures were maintained at 37 °C in a 5% CO_2_-humidified atmosphere for 7 days and sorted for purification by flow cytometry. For neutrophil–macrophage cocultures, neutrophils and macrophages (1:1) were mixed in the presence of 100 ng/ml LPS. After 6 h of culture, live cells were stimulated with LPS for intracellular cytokine staining and mRNA expression. For drug treatments, the cells were incubated with vehicle, Yoda1 (25 μM, MCE), ruthenium red (30 μM, Sigma), E6446 dihydrochloride (1 µM, Selleck), RU.521 (10 μM, Selleck), HTHQ (5 µM, MCE), GSK484 (5 µM, MCE) and DNase I (2.5 U/ml, Sigma) for 0.5–1 h before stimulation.

Flow cytometry was performed with the following antibodies from eBioscience, BD Biosciences, Biolegend or Abcam: anti-CD11b FITC (M1/70), anti-Ly6G PE (RB6-8C5), anti-F4/80 PE (BM8), anti-CD19 PE (1D3), anti-TCR FITC (H57-597), anti-CD11c FITC (N418), anti-CD45 APC (30-F11), anti-NK1.1 PE (PK136), anti-histone H3 (citrulline R2 + R8 + R17) (ab5103), anti-CD289-PE (TLR9), and anti-cGAS-PE (D-9).

FACS-based intracellular staining of cytokines was performed as previously described [33]. The cells were stimulated with LPS (100 ng/ml, Sigma–Aldrich, St. Louis, MO, USA) and GolgiPlug (BD Pharmingen, Lake Franklin, NJ, USA) for 5 h. BD Cytofix/Cytoperm and BD Perm/Wash buffer sets were used according to the manufacturer’s instructions (BD Pharmingen, Lake Franklin, NJ, USA). Anti-IL-10 (ICFC) and anti-tumour necrosis factor α (TNFα; MP6-XT22) antibodies were obtained from Biolegend (San Diego, CA, USA). Intracellular staining analysis was performed as previously described [33] using an anti-HIF-1α antibody (EPR16897; Abcam). Nonspecific FcR binding was blocked by the anti-mouse FcR mAb 2.4G2 (553142, BD Pharmingen). Nonviable cells were excluded via 7-AAD (555815, BD Pharmingen) staining. The percentage of cells stained with a particular reagent was determined by subtracting the percentage of cells stained non-specifically with the negative control mAb or Fluorescence Minus One (FMO) control from the percentage of those stained in the same dot-plot region as the anti-mouse mAbs. Flow cytometry data were acquired on a FACSCalibur (Becton Dickinson, CA, USA) or an Epics XL bench-top flow cytometer (Beckman Coulter, CA, USA), and the data were analysed with FlowJo (RRID:SCR_008520; Tree Star, San Carlos, CA, USA).

### Quantitative RT‒PCR

RNA was extracted with a RNeasy kit (QIAGEN, Dusseldorf, Germany), and cDNA was synthesized using SuperScript III reverse transcriptase (Invitrogen, Carlsbad, CA). An ABI 7900 real-time PCR system was used for quantitative PCR, with primer and probe sets obtained from Applied Biosystems (Carlsbad, CA). The results were analysed via SDS 2.1 software (Applied Biosystems). The cycling threshold value of the endogenous control gene (*Hprt*1, which encodes hypoxanthine guanine phosphoribosyl transferase) was subtracted from the cycling threshold. The expression of each target gene is presented as the fold change relative to that of the control samples.

### RNA sequences

Three parallel RNA samples of BALF cells from vehicle- or virus-infected mice were extracted with TRIzol reagent and used for RNA sequence analysis. Read quality was assessed for each sample using FastQC, and differential expression analysis was performed with DESeq2. The RNA sequence data analysis was completed by Novogen, Beijing, China. All of the data were deposited into the GEO series database under accession number GSE220198.

### Western blot

Sorted CD11b^+^Ly6G^+^ neutrophils or CD11b^+^F4/80^+^ macrophages from the BALF of mice were washed twice with cold PBS and lysed in RIPA buffer (50 mM Tris-HCl, pH 7.4; 1% NP-40; 0.25% Na-deoxycholate; 150 mM NaCl; and 1 mM EDTA, pH 7.4) for 10 min on a rocker at 4 °C. The protein concentration was determined via BCA (Beyotime, Shanghai, China). The protein samples were separated by 10% SDS‒PAGE and then transferred onto 0.22 µm polyvinylidene fluoride membranes (Merck Millipore, Bedford, MA, USA). The membranes were blocked with 5% nonfat dried milk for 1 h at room temperature and incubated with primary antibodies overnight on a shaker at 4 °C. Subsequently, an HRP-conjugated secondary antibody (Beyotime, Shanghai, China) was added for 1 h at room temperature. After washing, protein samples were detected via chemiluminescence (Merck Millipore, Bedford, MA, USA) using AllDoc-x software with a Tanon 5200 Imager (Tanon, Shanghai, China). The following primary Abs were used: anti-STING (D2P2F), anti-IRF3 (D83B9), and anti-SIRT2 (D4O5O) were obtained from Cell Signalling Technology (Danvers, MA, USA); anti-β-actin (AC-15) was obtained from Sigma‒Aldrich (St. Louis, MO, USA); anti-histone H3 (citrulline R2 + R8 + R17) (ab5103), anti-PAD4 [EPR20706] (ab214810) and anti-GAPDH [6C5] (ab8245) were obtained from Abcam (Cambridge, UK); and anti-Piezo1 polyclonal antibodies were purchased from Proteintech (Rosemont, USA).

### Statistical analysis

All of the data are presented as the means ± SDs. Student’s unpaired *t* test for parametric data or the Mann‒Whitney test for nonparametric data was used when two samples were compared, and one-way ANOVA with Dunnett’s post hoc test for parametric data or the Kruskal‒Wallis test for nonparametric data was used when more than two samples were compared. Differences between groups were considered statistically significant when the *P* value (alpha value) was less than 0.05.

## Results

### Neutrophil NET formation and macrophage differentiation are related to pulmonary respiratory virus infection

To observe the role of innate immune cells after acute respiratory virus infection, we established a PR8 influenza virus mouse infection model. After 48 h of virus infection, as the clinical symptoms gradually worsened, the ratio of dry weight to wet weight in the lungs of the mice decreased (Supplementary Fig. S1A), and more immune cells infiltrated. These infiltrating immune cells included mainly neutrophils and macrophages (Supplementary Fig. S1B-C). The infiltrating macrophages produced more TNFα and NOS2 and less IL-10 and CD206 (Supplementary Fig. S1D), which indicates that more M1 and less M2 macrophage differentiation are involved in antiviral immunity. Compared with other cells, infiltrating neutrophils produced more NETs (Supplementary Figs. S1E, S2A-B), ROS and CXCR2 (Supplementary Fig. S2C-D) and induced higher expression of PAD4 (Supplementary Fig. S2E), citrullinated histone H3 (Cit-H3) (Supplementary Fig. S3A-C). PAD4 and ROS are key regulators of NETs, and Cit-H3 is a component of NETs [3, 8]. Importantly, the percentage of neutrophil NETs was positively correlated with the number of infiltrating macrophages and the degree of M1 macrophage differentiation (Supplementary Fig. S3D-F). These data collectively suggest that neutrophil NET formation and macrophage differentiation are likely related to the outcome of the respiratory anti-viral response in mice.

### Piezo1 is critical for neutrophil NET formation in antiviral immunity

To understand how neutrophils and macrophages are regulated by virus infection, we compared the gene expression profiles of neutrophils from BALF from vehicle- and virus-infected mice via RNA sequencing and found that the expression patterns of select groups of surface and intracellular inflammatory signalling molecules, including chemokine receptors, cytokine receptors, and neutrophil granzymes, as described in Supplementary Fig. S4A, were altered in virus-infected neutrophils. Virus-infected neutrophils also presented upregulated expression of the ion channel receptor Piezo1. Piezo1 expression in neutrophils in virus-infected mouse lungs and BALF was upregulated (Supplementary Fig. S4B-C), which suggests that Piezo1 may be involved in regulating neutrophil function, including NET formation, in virus-infected mice.

We tested the application of a pharmacological approach to target Piezo1 and observed its effects on neutrophil function in the presence of a virus in vitro. Neutrophils were sorted from wild-type (WT) mouse spleens via flow cytometry and stimulated with virus in the presence or absence of Yoda1, an agonist of Piezo1. Compared with WT control neutrophils, Yoda1-treated neutrophils presented increased Piezo1 expression, increased NET and ROS production, and increased PAD4 expression (Supplementary Fig. S5A-D). These data collectively suggest that Piezo1 is sufficient for regulating neutrophil functions, including NET formation, in antiviral immunity.

### Piezo1 is required for neutrophil NET formation and macrophage differentiation during antiviral infection

**Fig. 1:**
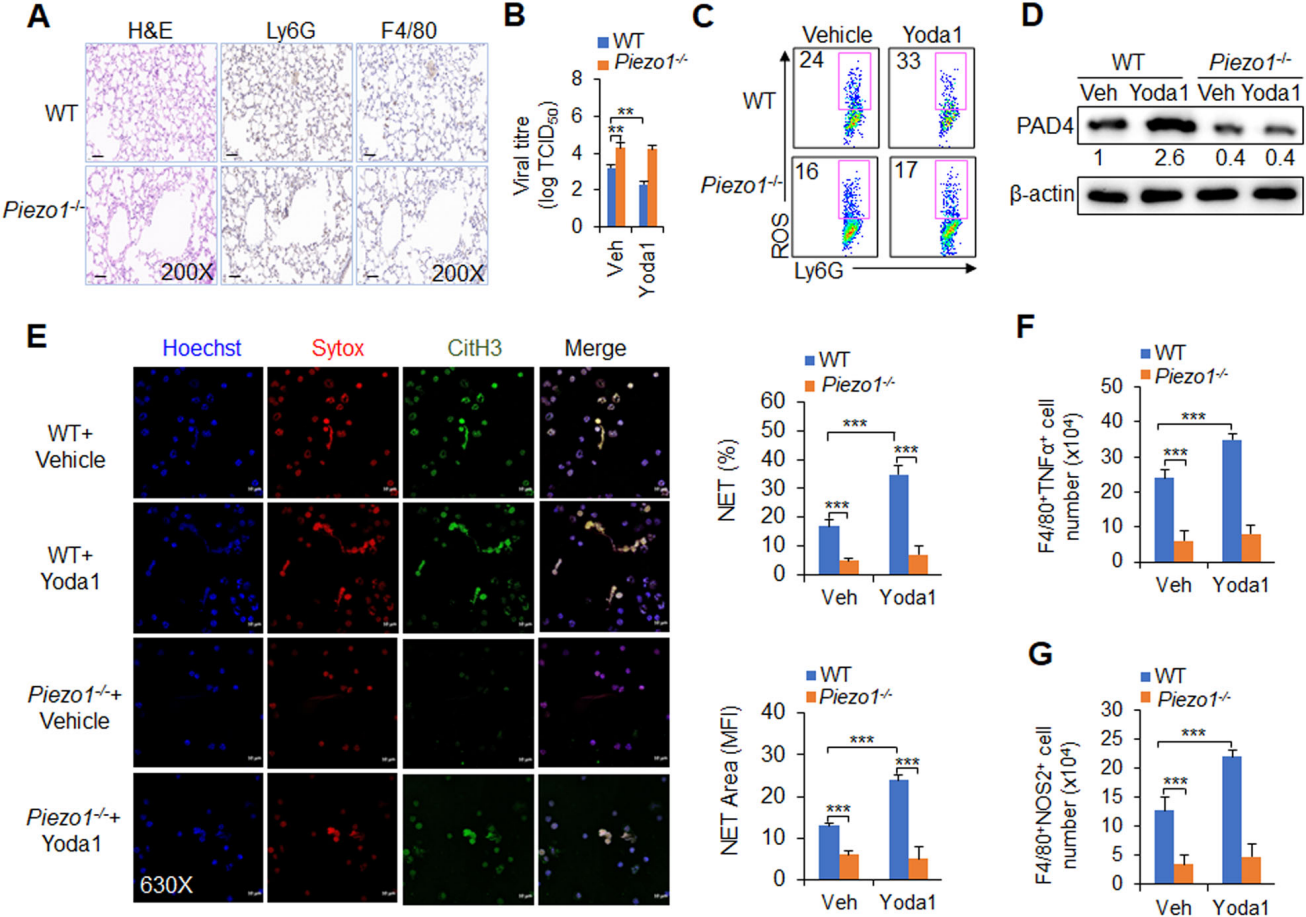
Piezo1 regulates neutrophil NET formation and macrophage differentiation during virus infection. Wild-type (WT) and *Piezo1*^−/−^ mice were infected with the PR8 virus for 48 h and treated with or without Yoda1 (2.6 mg/kg, MCE). **A** Haematoxylin and eosin (H&E) staining and immunohistochemical (IHC) staining of F4/80 (macrophage marker) and Ly6G (neutrophil marker) of infected mouse lungs. Scale bars, 10 µm; original magnification, 200X. **B** Lung virus titre of infected mice. TCID_50_, data are shown in log_10_ scale per lung lobe. **C** Expression of ROS in neutrophils isolated from BALF by flow cytometry. Dot plots present representative data. **D** Western blot analysis of PAD4 in neutrophils isolated from BALF. **E** NETs from neutrophils isolated from BALF were examined via confocal fluorescence microscopy. Typical NET images are displayed (left), and the percentage and area of NETs were quantified (right). Scale bars, 10 µm; original magnification, 630X. Intracellular staining of TNFα (**F**) and NOS2 (**G**) in CD11b^+^F4/80^+^ macrophages isolated from the BALF of virus-infected mice by flow cytometry. The graph shows data from three independent experiments with four mice per group. **P < 0.01 and ***P < 0.001, compared with the indicated groups.

Is Piezo1 necessary to induce neutrophil NET production and regulate macrophage differentiation during antiviral infection? We generated myeloid-specific *Piezo1* conditional knockout mice with *Piezo1*^flox/flox^ and *lysm-cre*, which are hereafter referred to as *Piezo1*^−/−^ mice. WT and *Piezo1*^−/−^ mice were infected with the PR8 virus and treated with or without Yoda1. We investigated the regulatory effect of Piezo1 on neutrophil and macrophage functions in virus-infected mice. Piezo1-deficient mice presented similar numbers of infiltrating neutrophils and macrophages in the BALF and lungs as WT mice following virus infection (Fig. 1A and Supplementary Fig. S5E). However, after virus infection, Piezo1 expression upregulation by Yoda1 treatment inhibited the virus load of 50% tissue culture infective dose (TCID_50_) at 48 h post infection; moreover, compared with WT control treatment, Yoda1 treatment mice presented increased PAD4 expression, ROS production and NET formation in neutrophils and increased TNFα and NOS2 expression in macrophages, whereas *Piezo1*^−/−^ mice presented opposite alterations and even abolished these effects of Yoda1 treatment in WT mice (Fig. 1B–F, Supplementary Figs. S5F, S6A-E). These data collectively suggest that Piezo1 is necessary for neutrophil function, including NET formation and M1 macrophage differentiation, during antivirus infection in mice.

### Piezo1-directed neutrophil NETs regulate macrophage differentiation during antiviral infection

Can neutrophil NETs direct the differentiation of macrophages? Inflammatory stimuli induce the formation of neutrophil NETs, which are further released to the extracellular environment of neutrophils. Thus, the effects of different stages of NET production on the regulation of macrophage differentiation were investigated via an in vitro coculture system involving neutrophils and macrophages. Bone marrow-derived macrophages (BMDMs) were obtained as described previously [9]. BM cells were treated with M-CSF for 7 days, and F4/80^+^ macrophages were sorted via flow cytometry. Neutrophils were sorted from the mouse spleen via flow cytometry, stimulated with LPS to induce the generation of NET DNA in vitro, and the supernatant (sup.), cells, or both were collected. Blocking NET DNA with DNase treatment in each group, including cells, sup., or both, led to decreased TNFα and NOS2 (M1 macrophages) (Supplementary Fig. S7A-C). These findings suggest that NET DNA produced by neutrophils, whether newly produced or released into the supernatant, can effectively promote M1 macrophage differentiation.

**Fig. 2:**
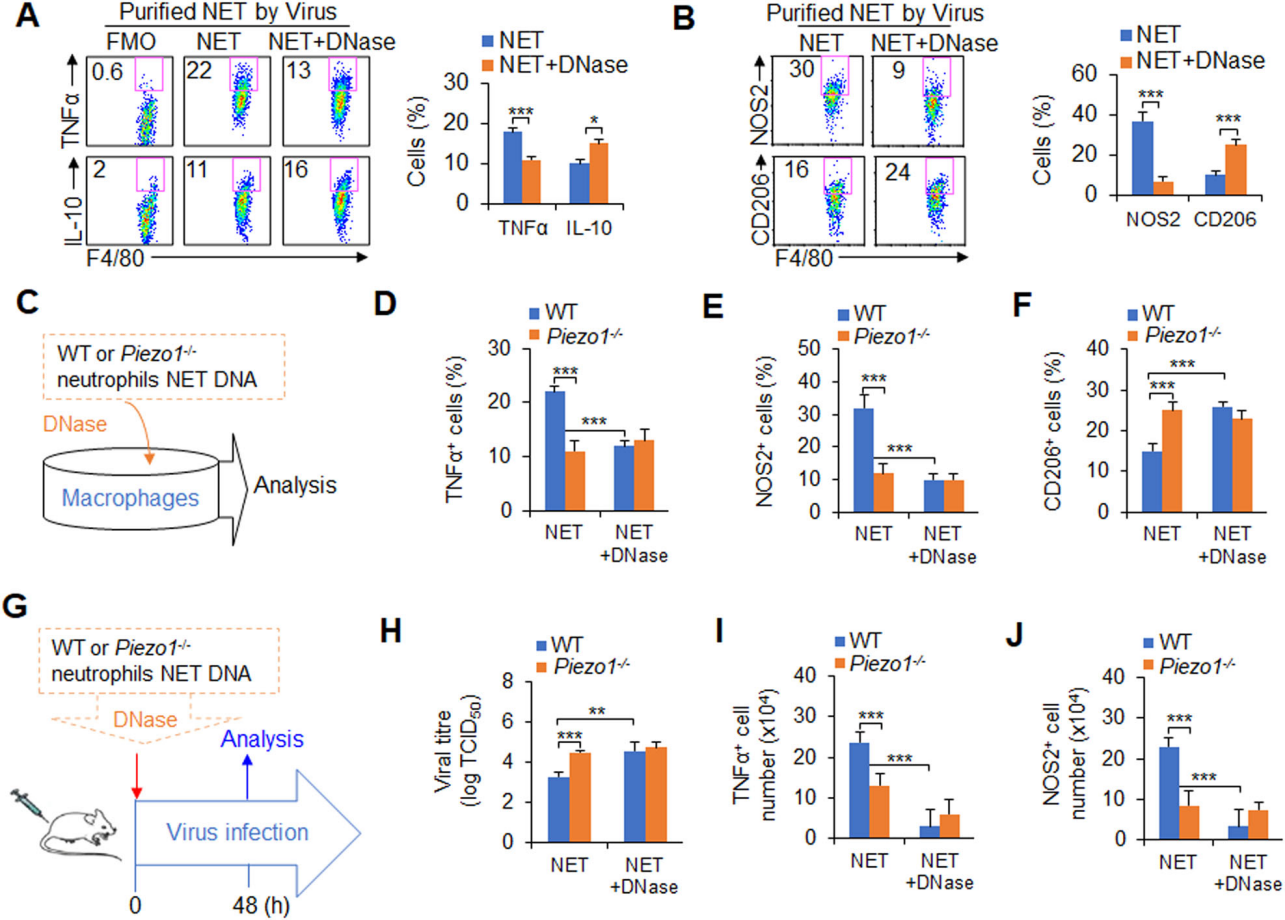
*Piezo1*^−/−^ neutrophil NET DNA inhibited M1 macrophage differentiation during the response to viral infection. **A**, **B** Neutrophils were isolated from WT mouse spleens and stimulated with the PR8 virus in vitro for 6 h, and NET DNA was purified and collected for subsequent experiments. Bone marrow-derived macrophages (BMDMs) were treated with NET DNA (1 ng/µl) from neutrophils for 6 h, and the levels of TNFα, IL-10 (**A**), NOS2 and CD206 (**B**) in the macrophages were determined via flow cytometry. Dot plots present representative data from flow cytometry analysis (left), and the statistical results are shown (right). Fluorescence Minus One control, FMO. **C**‒**F** Neutrophils (1 × 10^7^) were isolated from WT or *Piezo1*^−/−^ mouse spleens and stimulated with the PR8 virus in vitro for 6 h, after which NET DNA was purified and collected for subsequent experiments. BMDMs were treated with NET DNA extracted from the same number of WT or *Piezo1*^−/−^ neutrophils in the presence of virus for 6 h (**C**). Intracellular staining of TNFα (**D**), NOS2 (**E**) and CD206 (**F**) in macrophages was determined by flow cytometry. **G**–**J** Neutrophils were isolated from WT or *Piezo1*^−/−^ mouse spleens and stimulated with the PR8 virus in vitro for 6 h, after which NET DNA was purified and collected for subsequent experiments. NET DNA extracted from the same number of WT or *Piezo1*^−/−^ neutrophils was pretreated with or without DNase and i.v. injected into recipient mice. Simultaneously, the recipient mice were infected with the PR8 influenza virus for 48 h (**G**). **H** Lung virus titre of infected mice. TCID_50_, data are shown in log_10_ scale per lung lobe. Intracellular staining of TNFα (**I**) and NOS2 (**J**) in macrophages from BALF was determined by flow cytometry. The graph shows data from three independent experiments with three or four mice per group. *P < 0.05, **P < 0.01 and ***P < 0.001, compared with the indicated groups.

Next, the role of purified NET DNA from neutrophils induced by LPS in macrophage differentiation was studied. Consistently, blocking NET DNA with DNase caused less TNFα, NOS2 and more IL-10 and CD206 (Supplementary Fig. S7D-F), indicating that NET DNA from neutrophils is critical for inducing M1 macrophage differentiation. Similarly, purified NET DNA from neutrophils induced by viruses also had similar effects on macrophage differentiation (Fig. 2A, B). These data suggest that NET DNA produced by neutrophils induced by LPS or viruses is required for the induction of M1 macrophage differentiation.

How does Piezo1 regulate neutrophil NET formation and M1 macrophage differentiation after virus infection? The effects on macrophage differentiation of NET DNA purified from the same number of WT and *Piezo1*^−/−^ neutrophils induced by viruses were studied via a method similar to that described above (Fig. 2C). Although *Piezo1*^−/−^ neutrophil NETs did not affect the expression of CD54, CD80, CD86 or MHCII (Supplementary Fig. S8A-B), less NET DNA from Piezo1-deficient neutrophils resulted in less TNFα, NOS2 and more CD206 in macrophages (Fig. 2D–F). Moreover, blocking NET DNA with DNase treatment restored the WT level to the *Piezo1*^−/−^ level (Fig. 2D–F). However, *Piezo1*^−/−^ did not affect the expression of TNFα, IL-10, NOS2 or CD206 in macrophages (Supplementary Fig. S8C-D). Taken together, these data reveal that Piezo1 is required for NET DNA production in neutrophils for macrophage differentiation during virus infection.

To further test this hypothesis in vivo, we selected an adoptive transfer model in mice, as described in Fig. 2G. Purified NET DNA from the same number of neutrophils was isolated from WT or *Piezo1*^−/−^ mice and pre-treated with or without DNase. The NET DNA was transferred into the same WT recipient mouse. Simultaneously, the mice were infected with the PR8 influenza virus. Compared with WT control mice, recipient mice that received *Piezo1*^−/−^ neutrophil NET DNA presented greater virus loads (Fig. 2H) and lower TNFα and NOS2 production in macrophages (Fig. 2I-J). However, pre-treatment of these NET DNA samples with DNAse abolished these alterations (Fig. 2H–J). These data suggest that neutrophil NET DNA is required for M1 macrophage differentiation by Piezo1 during antiviral immunity in mice.

Is macrophage differentiation induced by neutrophil NETs induced by Piezo1 necessary for combating viral infection? Using clodronate liposomes to deplete macrophages in mice, we investigated the role of macrophage differentiation induced by *Piezo1*^−/−^ neutrophil NET DNA in viral infection in mice (Supplementary Fig. S9A). The results revealed that recipient mice that received *Piezo1*^−/−^ neutrophil NET DNA presented an increased virus load, an increased ratio of dry weight to wet weight in the lungs, and reduced TNFα and NOS2 production in macrophages (Supplementary Fig. S9B-E). Interestingly, treatment with clodronate liposomes abolished these alterations (Supplementary Fig. S9B-C). These data suggest that M1 macrophage differentiation induced by Piezo1 neutrophil NETs DNA is required for antiviral immunity in mice.

### Neutrophil NET DNA regulates M1 macrophage differentiation through TLR9 and cGAS signalling induced by Piezo1

**Fig. 3:**
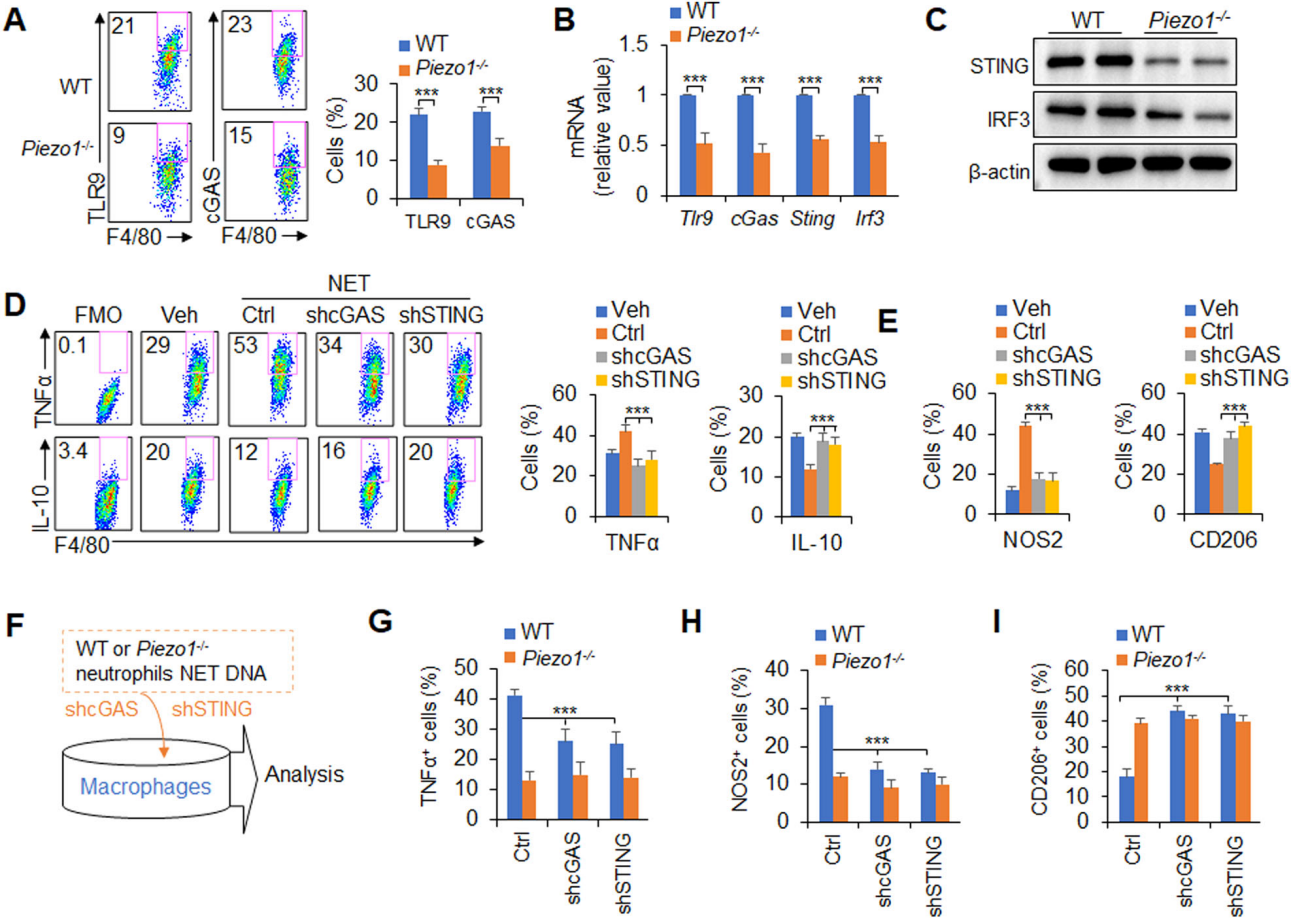
*Piezo1*^−/−^ neutrophil NET DNA inhibits M1 macrophage differentiation through TLR9 and cGAS signalling. **A**–**C** Wild-type (WT) and *Piezo1*^−/−^ mice were infected with the PR8 virus for 48 h. **A** TLR9 and cGAS expression in CD11b^+^F4/80^+^ macrophages in the BALF was measured via flow cytometry. Dot plots present representative data from flow cytometry analysis (left), and the statistical results are shown (right). **B** mRNA expression of the indicated genes in CD11b^+^F4/80^+^ macrophages sorted from BALF by flow cytometry. **C** Western blot analysis of STING and IRF3 in sorted CD11b^+^F4/80^+^ macrophages in the BALF via flow cytometry. **D**, **E** Neutrophils were isolated from mouse spleens and stimulated with virus in vitro for 6 h, and NET DNA was purified and collected for subsequent experiments. Bone marrow-derived macrophages were transfected with shRNA control (ctrl), shRNA-mediated cGAS (shcGAS) or shRNA-mediated STING (shSTING) and treated with NET DNA (1 ng/µl) from neutrophils stimulated with virus for 6 h. The levels of TNFα, IL-10 (**D**), NOS2 and CD206 (**E**) in macrophages were determined via flow cytometry. Fluorescence Minus One control, FMO. **F**–**I** Neutrophils of the same number were isolated from WT and *Piezo1*^−/−^ mouse spleens and stimulated with virus in vitro for 6 h, after which NET DNA was purified and collected for subsequent experiments. BMDMs were transfected with shRNA control (ctrl), shRNA cGAS (shcGAS) or shRNA STING (shSTING) and treated with NET DNA from WT and *Piezo1*^−/−^ neutrophils in the presence of virus for 6 h (**F**). Intracellular staining of TNFα (**G**), NOS2 (**H**) and CD206 (**I**) in macrophages was determined by flow cytometry. The graph shows data from three independent experiments with three to four mice per group. ***P < 0.001, compared with the indicated groups.

How do neutrophil NETs direct M1 macrophage differentiation? As reported previously [11, 13], TLR9 and cGAS are important DNA sensors of macrophages, and we first observed their expression in macrophages. Interestingly, after virus infection in mice, *Piezo1*^−/−^ mice presented reduced TLR9, cGAS, STING and IRF3 expression in macrophages in the BALF (Fig. 3A–C). These data suggest that the TLR9 and cGAS–STING–IRF3 signalling pathways are likely involved in neutrophil NET DNA-directed M1 macrophage differentiation induced by Piezo1.

To test this hypothesis, we applied a pharmacological approach to target TLR9 and cGAS to observe their effects on macrophage differentiation in vitro. The role of purified NET DNA from neutrophils in mice induced by viruses in terms of macrophage differentiation was studied. Although the purified NET DNA from neutrophils induced more NOS2 and TNFα and less CD206 and IL-10 in macrophages, blocking TLR9 and cGAS with their inhibitors reversed these alterations (Supplementary Fig. S10A-C). These data suggest that the TLR9 and cGAS pathways are likely involved in regulating macrophage differentiation induced by NET DNA of neutrophils.

Consistent with these findings, knockdown of cGAS and STING with shRNA in macrophages resulted in similar alterations. The purification of NET DNA from neutrophils infected with the virus increased the levels of NOS2 and TNFα and decreased the levels of CD206 and IL-10 in macrophages, and the knockdown of cGAS and STING reversed these alterations (Fig. 3D, E). Taken together, these data suggest that the cGAS–STING signalling pathway is required for neutrophil NET DNA-induced M1 macrophage differentiation during virus infection, and we further hypothesized that Piezo1 plays a regulatory role in antiviral immunity.

To test this hypothesis, we knocked down cGAS and STING with shRNA in macrophages and observed the role of macrophage differentiation induced by NET DNA from *Piezo1*^−/−^ neutrophils (Fig. 3F). Although less NET DNA from *Piezo1*^−/−^ neutrophils than WT control neutrophils induced less expression of TNFα and NOS2 and more expression of IL-10 and CD206 in macrophages, knockdown of cGAS or STING reversed these alterations (Fig. 3G–I), indicating that cGAS-STING is required for M1 macrophage differentiation induced by NET DNA of *Piezo1*^−/−^ neutrophils during the response to virus infection.

### Piezo1 directs neutrophil NET formation though SIRT2-HIF1α signalling during virus infection

What regulates neutrophil NET production during virus infection? As reported [34, 35], PAD4 and ROS are critical for regulating NET formation in neutrophils during inflammation. Blocking PAD4 with shRNA (Supplementary Fig. S11A-C) restored the ROS level and NET formation in neutrophils, indicating that ROS and NET formation are dependent on PAD4 signalling in neutrophils during virus infection. Consistently, blocking ROS with inhibitor treatment reduced the production of NETs but did not alter the expression of PAD4 in neutrophils (Supplementary Fig. S11D-F). These data suggest that ROS are required for PAD4-mediated NET formation in neutrophils during the response to virus infection.

**Fig. 4:**
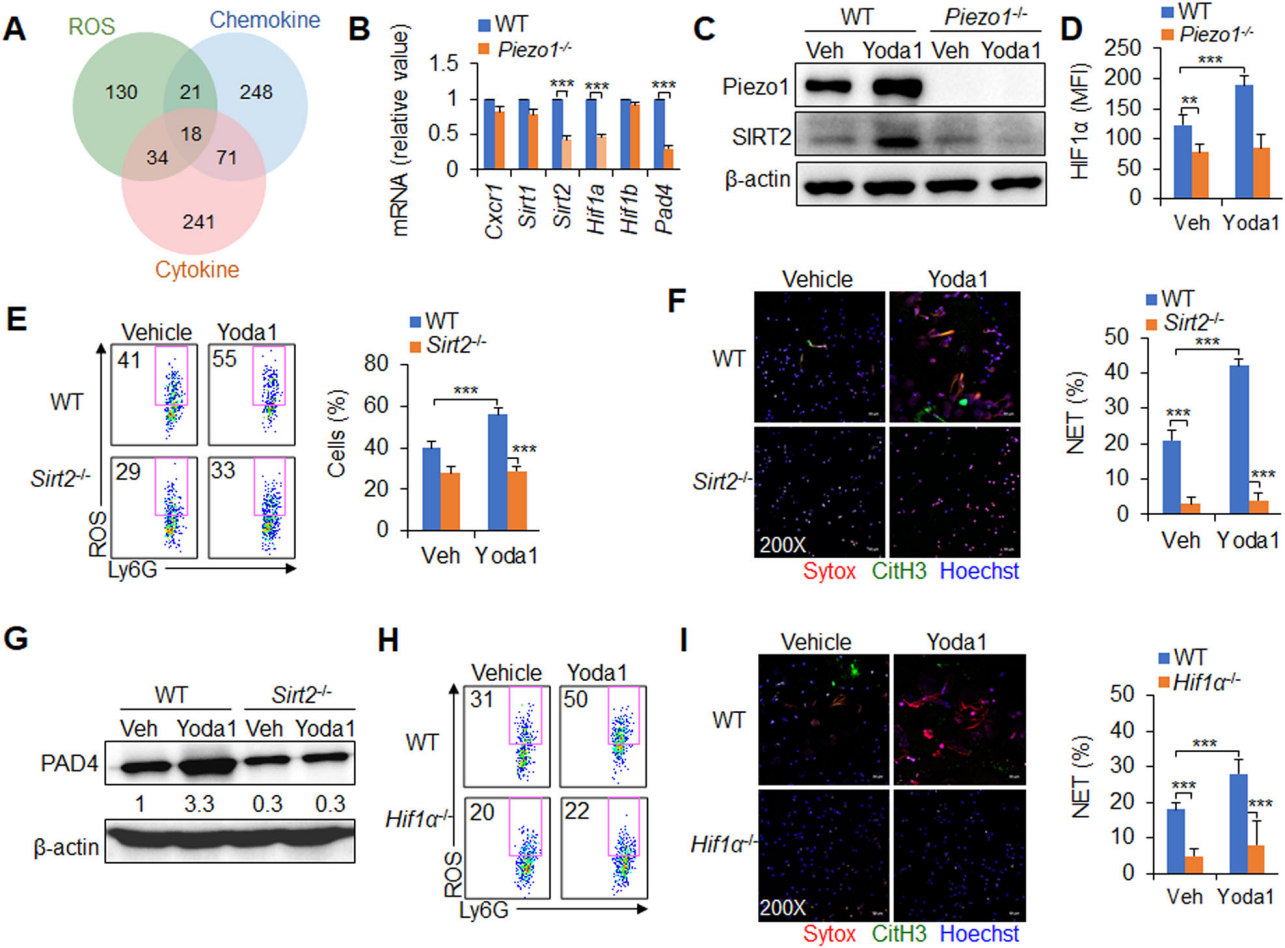
Piezo1 regulates neutrophil NET formation though SIRT2-HIF1α signalling during virus infection. **A** C57BL/6 mice were infected with the PR8 virus for 48 h, and the lungs were collected. RNA was analysed via RNA sequencing to compare the expression profiles of control and virus-infected cells from the lung with those of certain genes involved in the ROS, inflammatory cytokine and chemokine signalling pathways. **B**–**D** Wild-type (WT) and *Piezo1*^−/−^ mice were infected with the PR8 virus for 48 h and treated with or without Yoda1 (2.6 mg/kg), as indicated. **B** mRNA expression of the indicated genes in neutrophils isolated from BALF. **C** Western blot of Piezo1 and SIRT2 in sorted neutrophils from BALF. **D** Intracellular staining of HIF1α in neutrophils isolated from BALF. **E**–**G** WT and *Sirt2*^−/−^ mice were infected with the PR8 virus for 48 h and treated with or without Yoda1 (2.6 mg/kg). **E** Expression of ROS in neutrophils isolated from BALF. Dot plots present representative data from flow cytometry analysis (left) and summarized data (right). **F** NET formation in neutrophils sorted from BALF by confocal fluorescence microscopy. Typical NET images are displayed (left), and the percentage of NETs was quantified (right). Scale bars, 50 µm; original magnification, 200X. **G** Western blot of PAD4 in sorted neutrophils from BALF. **H**, **I** WT and *Hif1α*^−/−^ mice were infected with the PR8 virus for 48 h and treated with or without Yoda1 (2.6 mg/kg). **H** Expression of ROS in neutrophils isolated from BALF by flow cytometry, and dot plots present representative data from flow cytometry analysis. **I** NET formation in neutrophils sorted from BALF by confocal fluorescence microscopy. Typical NET images are displayed (left), and the percentage of NETs was quantified (right). Scale bars, 50 µm; original magnification, 200X. The graph shows data from three independent experiments with four mice per group. **P < 0.01 and ***P < 0.001, compared with the indicated groups.

How does Piezo1 regulate neutrophil NET formation during antivirus infection? ROS, inflammatory cytokines and chemokines are important in the formation of neutrophil NETs and are regulated by Piezo1. To screen the key molecules that play an integrated regulatory role downstream of Piezo1, we analysed the neutrophil data of virus infection via RNA sequencing and identified 18 target molecules in the integrated region of the three regulatory-related signalling pathways (Supplementary Fig. S12A and Fig. 4A). mRNA expression analysis of these molecules revealed that the deletion of Piezo1 reduced the mRNA expression of *Sirt2*, *Hif1a* and *Pad4* but not that of *Cxcr1*, *Sirt1* and *Hif1β* in neutrophils (Fig. 4B). *Piezo1*^−/−^ or Piezo1 upregulation by Yoda1 treatment altered SIRT2 and HIF1α expression in neutrophils (Fig. 4C, D), indicating that SIRT2 and HIF1α are likely involved in Piezo1-mediated NET formation in neutrophils during the response to virus infection.

To investigate the role of SIRT2 in Piezo1-directed neutrophil NET formation, we generated myeloid-specific SIRT2 conditional knockout mice with *Sirt2*^flox/flox^ and *lysm-cre*, and these mice are referred to as *Sirt2*^−/−^ mice hereafter. *Sirt2*^−/−^ reduced PAD4 expression, ROS production and NET formation in neutrophils. Although Piezo1 expression upregulation by Yoda1 treatment increased PAD4 expression, ROS production, and NET formation, *Sirt2*^−/−^ cells treated with Yoda1 reversed these alterations, but not Piezo1 expression (Fig. 4E–G, Supplementary Figs. S12B-E, S13). These findings suggest that SIRT2 is required for regulating the formation of NETs downstream of Piezo1 during the response to virus infection.

Similarly, to investigate the role of HIF1α in Piezo1-directed neutrophil NET formation, we generated myeloid-specific HIF1α conditional knockout mice with *Hif1α*^flox/flox^ and *lysm-cre*, and these mice are called *Hif1α*^−/−^ mice hereafter. *Hif1α*^−/−^ reduced PAD4 expression, ROS production and NET formation in neutrophils. Although Piezo1 expression upregulation by Yoda1 treatment increased PAD4 expression, ROS production and NET formation, *Hif1α*^−/−^ cells treated with Yoda1 reversed these alterations, but not Piezo1 and SIRT2 expression (Fig. 4H–I, Supplementary Figs. S14A-F, S15A). These findings suggest that HIF1α is required for regulating the formation of NETs in neutrophils downstream of Piezo1–SIRT2 during the response to virus infection.

### Magnesium is sufficient for Piezo1-mediated NET formation in the response to virus infection

**Fig. 5:**
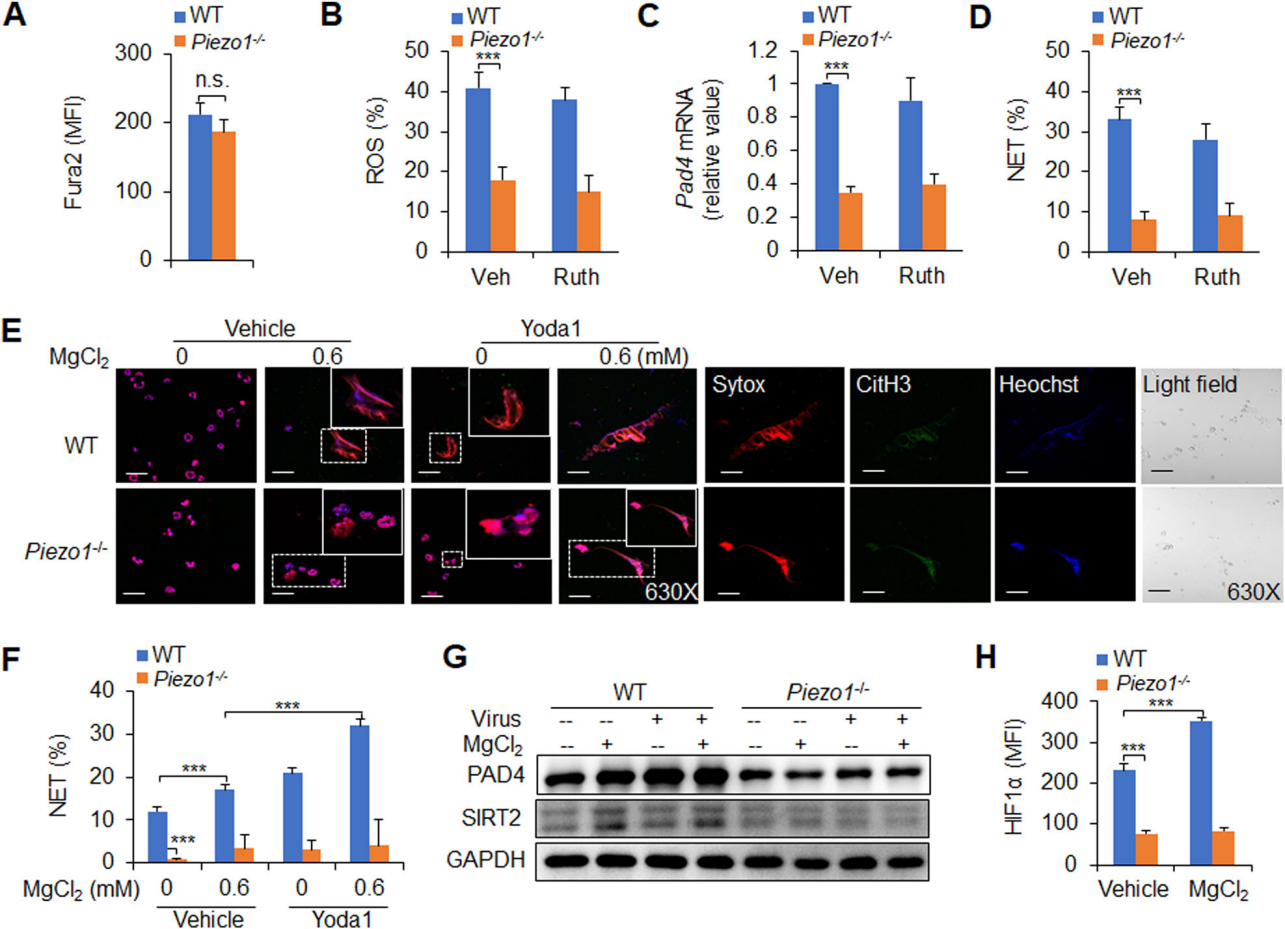
Magnesium-sensing Piezo1-directed NET formation in neutrophils during antivirus infection. **A** Wild-type (WT) or *Piezo1*^−/−^ mice were infected with the PR8 virus for 48 h, and the BALF was collected. Measurement of intracellular Ca^2+^ concentrations in neutrophils with Fura2 dye. **B**–**D** Neutrophils isolated from the spleens of WT or *Piezo1*^−/−^ mice were stimulated with the virus for 6 h with or without ruthenium red (Ruth, 30 µm, Sigma). **B** Intracellular staining of ROS in neutrophils by flow cytometry and data summary. **C** *Pad4* mRNA expression in neutrophils by qPCR. **D** NET formation by sorted neutrophils from the spleen was analysed via confocal fluorescence microscopy, and the percentage of NETs was determined. **E**, **F** NET formation by confocal fluorescence microscopy in the spleen of sorted neutrophils from WT or *Piezo1*^−/−^ mice in the presence of virus for 6 h with or without MgCl_2_ (0 and 0.6 mM) and/or Yoda1 (25 μM, MCE). Typical NET images are displayed (**E**), and the percentage of NETs was quantified (**F**). Scale bars, 50 µm; original magnification, 630X. Western blot of PAD4 and SIRT2 (**G**) and intracellular staining of HIF1α (**H**) in sorted neutrophils from the spleens of WT or *Piezo1*^−/−^ mice stimulated with virus for 6 h with or without MgCl_2_ (0.6 mM). The graph shows data from three independent experiments with three to four mice per group. ***P < 0.001, compared with the indicated groups. n.s. not significant.

Piezo1 is a nonselective ion channel with significant permeability to calcium ions [36], and we first assessed the level of calcium influx in *Piezo1*^−/−^ splenic neutrophils. Compared with WT control neutrophils, *Piezo1*^−/−^ neutrophils presented comparable calcium influx in virus-infected mice (Fig. 5A). Although *Piezo1*^−/−^ cells reduced neutrophil ROS production, PAD4 expression and NET formation, blocking the calcium influx signalling pathway with ruthenium red, a calcium influx inhibitor, did not affect these alterations (Fig. 5B–D). These data suggest that calcium influx is likely not involved in Piezo1-mediated NET formation in neutrophils during the response to virus infection.

The magnesium ion has been shown to play an important role in antitumour immunity [37], but its role in antiinfection immunity is still unclear. To investigate the role of magnesium ions in the formation of neutrophil NETs, a magnesium-free medium culture system was used, and different concentrations of MgCl_2_ (0.6 mM is the physiological concentration) were added to observe NET formation and related molecular expression alterations in neutrophils. Under normal and viral infection conditions, magnesium at a physiological concentration (0.6 mM) increased NET formation and the expression of PAD4, SIRT2 and HIF1α; upregulation of Piezo1 expression by Yoda1 treatment promoted these effects, but *Piezo1*^−/−^ abolished these alterations (Fig. 5E–H). These data suggest that magnesium influx is sufficient for Piezo1 to induce neutrophil NET formation during the response to virus infection.

### Magnesium-sensing Piezo1-directed NETs regulate M1 macrophage differentiation during the response to viral infection in mice and humans

**Fig. 6:**
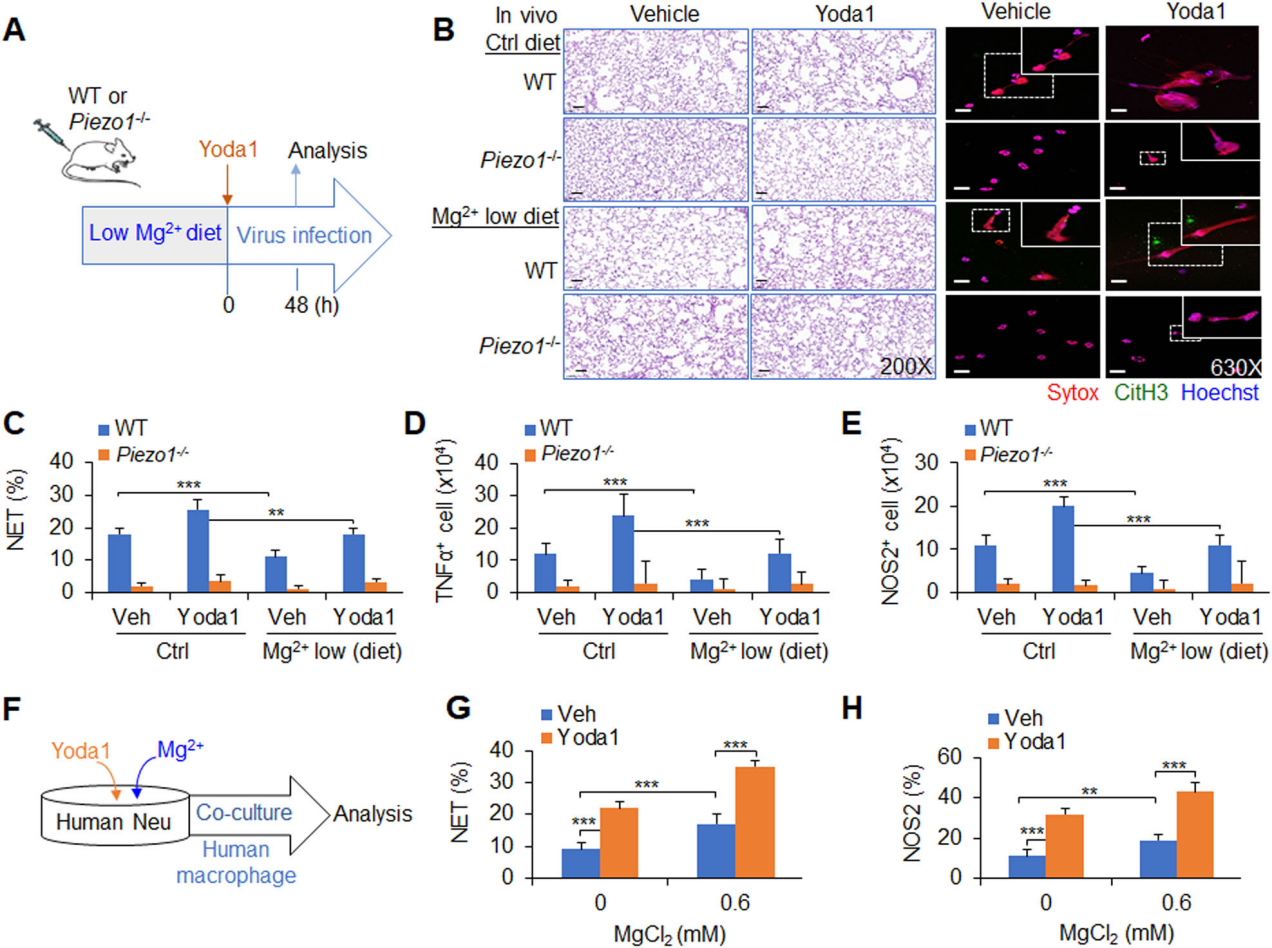
Magnesium-sensing Piezo1-directed NETs regulate M1 macrophage differentiation during viral infection in mice and humans. **A**–**E** Wild-type (WT) or *Piezo1*^−/−^ mice were infected with the PR8 virus for 48 h and treated with or without Yoda1 (2.6 mg/kg, MCE) under normal or low-magnesium diet conditions (**A**). Haematoxylin and eosin (H&E) staining of infected mouse lungs (**B**, left). NET formation in neutrophils were isolated from the BALF of WT or *Piezo1*^−/−^ mice via confocal fluorescence microscopy. Typical NET images are displayed (**B**, right), and the percentage of NETs was quantified (**C**). Scale bars, 50 µm; original magnification, 630X. Intracellular staining of TNFα (**D**) and NOS2 (**E**) in macrophages from the BALF of WT or *Piezo1*^−/−^ mice by flow cytometry, and the data are summarized. **F**–**H** Human CD34^+^ haematopoietic stem cells stimulated with G-CSF for 7 days were used as human neutrophils. Human peripheral blood CD14^+^ monocytes were stimulated with M-CSF for 7 days to generate human macrophages. A coculture system with neutrophils and macrophages was set up (**F**). **G** Neutrophils infected with virus in the absence or presence of magnesium (0.6 mM) and/or Yoda1 (25 µM, MCE) and NET formation in neutrophils were analysed via confocal fluorescence microscopy, and the percentage of NETs was quantified. **H** Purified NET DNA from human neutrophils was added to the macrophage culture for 12 h. Intracellular staining of NOS2 in macrophages by flow cytometry. The graph summarizes data from three independent experiments with three to four mice or samples per group. ** *P* < 0.01 and ***P < 0.001, compared with the indicated groups.

To test the significance of magnesium-sensing Piezo1-mediated NET formation in the response to anti-virus infection, WT or *Piezo1*^−/−^ mice were fed a low-magnesium diet and pre-treated with Yoda1 before PR8 virus infection. Piezo1 expression upregulated by Yoda1 treatment worsened local lung inflammation, and increased neutrophil NET formation and macrophage TNFα and NOS2 production. As expected, *Piezo1*^−/−^ abolished these alterations. Importantly, a low-magnesium diet weakened lung inflammatory changes, NET formation by neutrophils and TNFα and NOS2 production by macrophages (Fig. 6A–E and Supplementary Fig. S15B), suggesting that magnesium diet regulation is likely a helpful approach for regulating Piezo1-directed NET formation and macrophage differentiation during the response to viral infection.

Next, we tested the application of a pharmacological approach to target Piezo1 in human neutrophils and determined whether we can recapitulate our findings in the context of genetic targeting of Piezo1. We applied the Piezo1 agonist Yoda1 to human neutrophils, which are derived from human CD34^+^ haematopoietic stem cells induced by G-CSF for 7 days, and induced increased NET formation with or without magnesium. Interestingly, magnesium ions strengthened these effects (Fig. 6F, G). Furthermore, we applied Yoda1 treatment to a human neutrophil‒macrophage coculture system in which macrophages from human peripheral blood were induced to differentiate into CD14^+^ monocytes by M-CSF for 7 days. The pharmacological activation of Piezo1 in human neutrophils largely recapitulated what we observed in genetic mouse neutrophils in terms of the production of NOS2 in human macrophages. Moreover, magnesium could sensitize these effects (Fig. 6H). Thus, our data demonstrated the ability of magnesium-sensing Piezo1 to mediate an evolutionally conserved signalling pathway in mouse and human neutrophils.

**Fig. 7:**
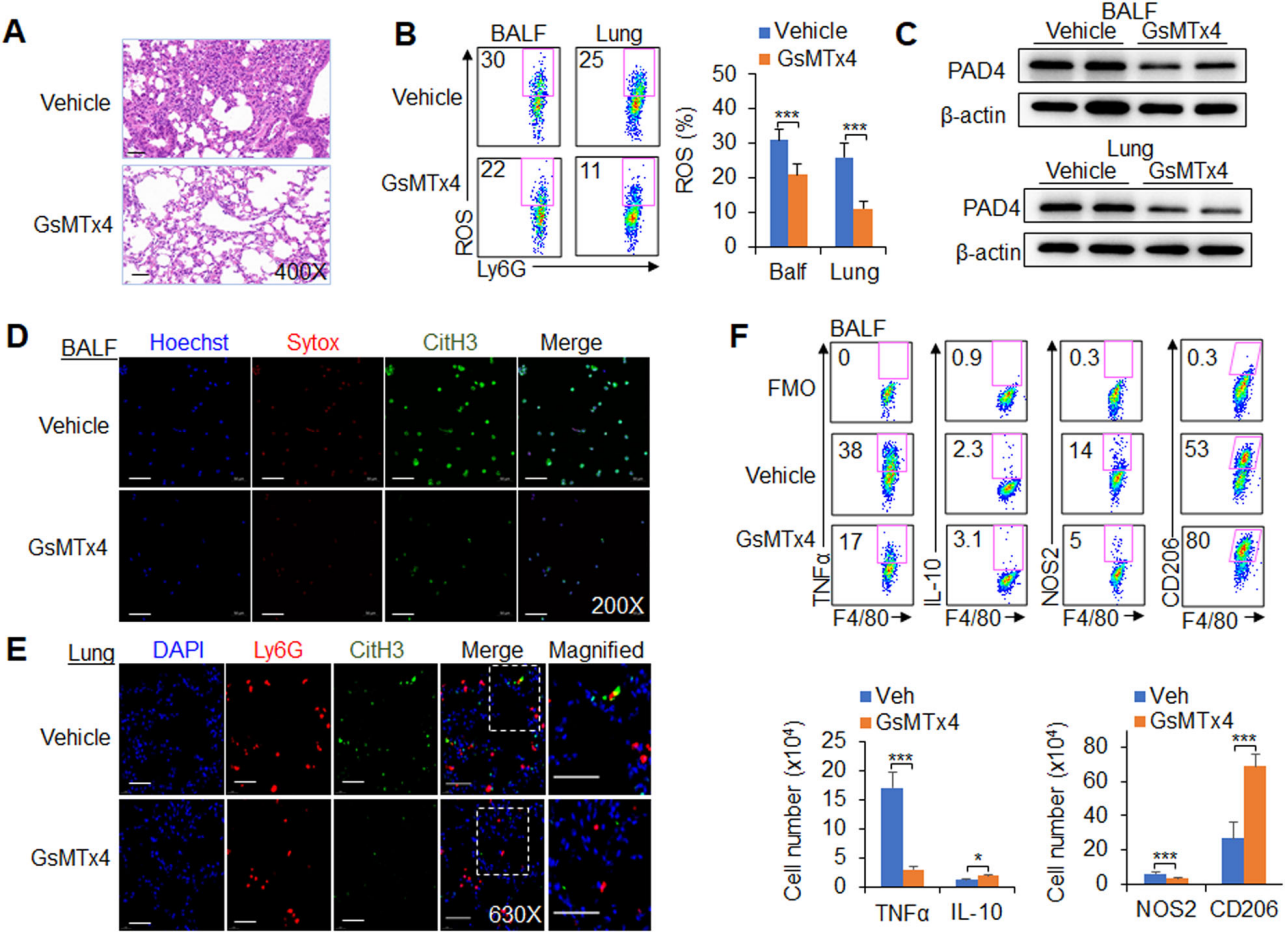
Piezo1 is necessary for neutrophil NET formation and macrophage differentiation during virus infection. Wild-type (WT) cells were infected with the PR8 virus for 48 h and treated with or without GsMTx4 (2.0 mg/kg, MCE). **A** Haematoxylin and eosin (H&E) staining of infected mouse lungs. **B** Expression of ROS in neutrophils isolated from the BALF and lungs by flow cytometry. Dot plots present representative data (left) and a summary of the data (right). **C** Western blot analysis of PAD4 in neutrophils isolated from the BALF and lungs. NETs from neutrophils isolated from the BALF (**D**) and lungs (**E**) were detected via confocal fluorescence microscopy. Typical NET images are displayed. Scale bars, 50 µm; original magnification, 630X. **F** Intracellular staining of TNFα, IL-10 and NOS2 and the expression of CD206 in CD11b^+^F4/80^+^ macrophages isolated from the BALF of virus-infected mice were measured via flow cytometry. Fluorescence Minus One control, FMO. Dot plots present representative data from flow cytometry analysis (upper), and the statistical results are shown (lower). The graph shows data from three independent experiments with four mice per group. *P < 0.05 and ***P < 0.001, compared with the indicated groups.

Finally, we tested the application of a pharmacological approach to target Piezo1 in neutrophils and determined whether our findings recapitulated our previous findings derived from the genetic targeting of Piezo1 in mice during viral infection. The mice were infected with the PR8 virus and treated with or without GsMTx4, an antagonist of Piezo1. We investigated the regulatory effect of Piezo1 on neutrophil and macrophage functions in virus-infected mice. After virus infection, Piezo1 channel activation was downregulated by GsMTx4 treatment, leading to reduced PAD4 expression, ROS production and NET formation in neutrophils, and TNFα and NOS2 expression was decreased in macrophages from the BALF and lung (Fig. 7A–F and Supplementary Fig. S16A-C). These data collectively suggest that targeting Piezo1 is effective for modulating neutrophil function, including NET formation and M1 macrophage differentiation, during the response to virus infection.

## Discussion

Neutrophils are critical for initiating first-line innate immune cells and subsequently inducing other innate and adaptive immune responses to protect against virus infections [2, 3]. During pathogenic microorganisms invasion, neutrophils are recruited to the site of inflammation to eliminate pathogenic microorganisms [7]. In addition, neutrophils are also able to transmit some antigen information to other immune cells by some means, inducing an immune response cascade and triggering a protective immune response [38, 39]. In this study, the ion sensor Piezo1 was shown to direct neutrophil NET formation and regulate M1 macrophage differentiation during the response to in influenza virus infection. *Piezo1*^−/−^ neutrophils had reduced NET DNA production, leading to decreased TLR9 and cGAS–STING signalling activity while inducing reciprocal differentiation from M1 to M2 macrophages. In addition, Piezo1 integrated magnesium signalling and the SIRT2–HIF1α signalling pathway to orchestrate reciprocal M1 and M2 macrophage lineage commitment through neutrophil NET formation (Supplementary Fig. S17). Thus, our studies provide comprehensive insight into the role of neutrophil-based mechanical regulation of immunopathology in directing macrophage lineage commitment during the response to influenza virus infection.

When pathogens such as respiratory influenza viruses invade the body, neutrophils are the first-line defenders recruited to the lesion loci of local respiratory tract and lungs. The immune functions of neutrophils include phagocytosis, the production of ROS, and the formation and release of NETs. Recent studies have shown that NETs are critically involved in defending against pathogen infection-induced inflammation and tumour inflammation. NETs, which are composed of deconcentrated chromatin DNA, the histone myeloperoxidase and neutrophil elastase, play important roles in defending against pathogenic microbial invasion [6, 7]. The release of NETs and their component extracellular DNA is an effective weapon for the immune response. As one of the innate immune components, neutrophils release NETs in response to invading pathogens, which help them capture and kill pathogenic microorganisms [6, 7]. However, it is still unclear whether neutrophil NETs have other regulatory effects. Our study revealed that neutrophil NETs are critical for inducing macrophage differentiation during antiviral immune responses. The release of NET DNA from neutrophils effectively triggers DNA sensor, TLR9 and cGAS–STING signalling activities to induce M1 macrophage differentiation in antiviral immunity.

The mechanism of neutrophil NET production has long been a focus of research. It has been shown that ROS are critical for NET formation in neutrophils. ROS can directly activate PAD4 to deconcentrate chromatin [40]. ROS can also release NE from azurophilic granules into the cytoplasm by activating MPO, where NE can bind to F-actin and degrade it to enter the nucleus [4, 5]. In the nucleus, NE can hydrolyse histones and destroy chromatin packaging, thus affecting the formation of NETs [41]. Consistent with previous studies, our study revealed that ROS and PAD4 are critically involved in regulating neutrophil NET formation during antiviral responses. The activation of NADPH oxidase complexes through the classical protein kinase C (PKC) or RAF-MEK-MAPK pathways results in the production of ROS and then the formation of NETs [42]. Our data further revealed that the NAD^+^-dependent deacetylase SIRT2, no other SIRT family members, is critically involved in regulating NET formation induced by Piezo1. The transcription factor HIF1α is critically involved in regulating Piezo1-induced innate immune cell function [18]. SIRT1 is responsible for the direct deacetylation and destabilization of HIF1α [43, 44]. Our data showed that SIRT2–HIF1α, a redox-related mechanism, is required for regulating PAD4–ROS signalling-mediated NET formation in *Piezo1*^−/−^ neutrophils during the immune response to viral infections.

Recent studies have reported that Piezo1 is involved in regulating the functions of various immune cells, including macrophages, DCs and T cells, in infectious inflammation and cancer. Piezo1 regulates macrophage polarization and stiffness sensing for macrophage activation [45]. The Piezo1-mediated macrophage inflammatory response to LPS occurs through the TLR4 pathway [36]. DC Piezo1 directs reciprocal differentiation between T_H_1 and regulatory T cells [21]. However, the regulatory role of Piezo1 in neutrophils has not been reported. Our data showed that influenza virus infection initiated Piezo1 activity and directed neutrophil NET formation. The ion channel Piezo1, as a signal node, senses the magnesium concentration and integrates the downstream SIRT2–HIF1α signalling pathway to direct NET formation during the response to viral infection.

Piezo1 was originally identified as a nonselective cation ion channel with significant permeability to calcium ions [45]. However, blocking calcium influx cannot affect Piezo1-mediated NET formation in neutrophils. It has been shown that magnesium-sufficiency sensed via LFA-1 is related to the superior performance of pathogen- and tumour-specific T cells, enhanced the effectiveness of bispecific T-cell-engaging antibodies, and improved CAR T-cell function [37]. Our data further revealed that magnesium-sufficiency is involved in Piezo1-directed NET formation to regulate macrophage differentiation during the response to virus infection. A low-magnesium diet ameliorates Piezo1-mediated NET formation and M1 macrophage differentiation, which contributes to relieving inflammation caused by virus infection. These data should be helpful for the dietary management of clinical respiratory tract virus infections.

Inflammatory signals and ion signals are closely associated with the immune response, but few studies have investigated the regulation of the magnesium signalling pathway and immune response. Our data showed that Piezo1 could respond to magnesium signalling pathways and further modulate neutrophil NET formation in the context of infectious inflammation. Effective immune responses require neutrophils to function under various conditions, including altered extracellular inflammatory states and ion levels (possibly caused by inflammatory stimulation) or nutritional and/or hypoxic environments (inflammatory microenvironments). The adaptation of neutrophils to changing redox states results from a “ion channel sensor checkpoint”, an active signalling process involved in sensing changes in extracellular inflammatory and magnesium levels and subsequent signal transduction and execution. Our data further suggested that the ion channel sensor Piezo1 in neutrophils is critically required for regulating NET formation and subsequent M1 macrophage differentiation.

In summary, targeting the ion channel sensor Piezo1 in neutrophils alters NET DNA production and TLR9 and cGAS signalling activities, thereby contributing to the reciprocal differentiation of M1 and M2 macrophages while responding to viral infection. Thus, our results define the essential nature of Piezo1 as a signalling node that transduces virus infectious signals and magnesium signals to initiate the signalling activities of the Piezo1–SIRT2-HIF1α signalling pathway in neutrophils for NET formation. This signalling induces the reciprocal differentiation of M1 and M2 macrophages and has implications for targeting neutrophils as an approach to the treatment of viral infections.

## Supplementary information

Supplementary Figures

Original Data

## Data Availability

The data that support the findings are available upon request to the corresponding author (Y.B. and G.L.). RNA-sequence data are available on Gene Expression Omnibus (GEO accession number GSE220198).
