# Supplementary material for: Piezo1-directed neutrophil extracellular traps regulate macrophage differentiation during influenza virus infection
Source: Cell Death Dis. 2025 Jan 31;16(1):60. doi: 10.1038/s41419-025-07395-5 (PMC11785962; doi:10.1038/s41419-025-07395-5)
Supplement: Supplementary file 1 — Supplementary Figures [file 41419_2025_7395_MOESM1_ESM.pdf]

Piezo1-directed neutrophils extracellular traps regulate macrophage differentiation during influenza virus infection

Yuexin Wang[1,#], Qiuli Yang[1,#], Yingjie Dong[1,#], Likun Wang[2,#], Zhiyuan Zhang[1], Ruiying Niu[1], Yufei Wang[1], Yujing Bi[2,†], Guangwei Liu[1,†]

[1]Key Laboratory of Cell Proliferation and Regulation Biology, Ministry of Education, College of Life Sciences, Beijing Normal University, Beijing 100875 China; [2]State Key Laboratory of Pathogen and Biosecurity, Academy of Military Medical Science, Beijing 100080 China

Supp. Fig.1

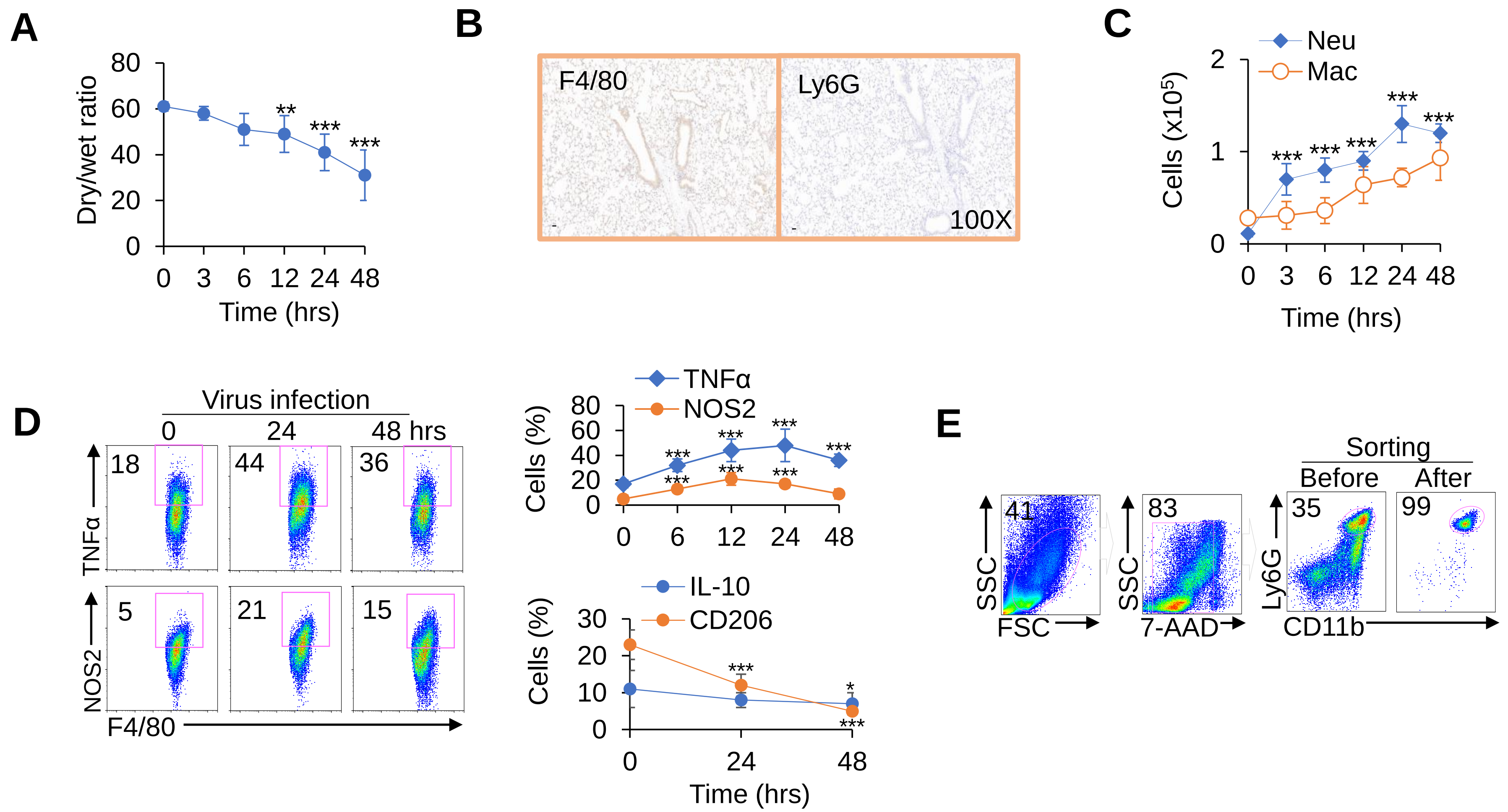

Fig. S1. Neutrophil NETs and macrophage differentiation are related with pulmonary respiratory virus infection.

(**A-D**) C57BL/6 mice challenged by PR8 virus infection at different time. (A) Ratio of dry to wet weight of lungs from the infected mice. (B) Immunohistochemical (IHC) staining of F4/80 (macrophage marker) and Ly6G (neutrophil marker) in lungs of infected mice. (C) Absolute number of $CD11b^{+}Ly6G^{+}$ neutrophils and $CD11b^{+}F4/80^{+}$ macrophages in BALF from infected mice. (D) Intracellular staining of TNFα, IL-10, and NOS2 and expression of CD206 in macrophages from BALF of infected mice by flow cytometry. Dot-plots present the representative data from flow cytometry analysis (left), and statistical results are shown (right). (**E**) The gating strategy and purity of sorted neutrophils from BALFs in mice. The graph summarizes data from three independent experiments with six to eight mice per group. **P*<0.05, ***P*<0.01 and ****P*<0.001, compared with the indicated groups.

Supp. Fig.2

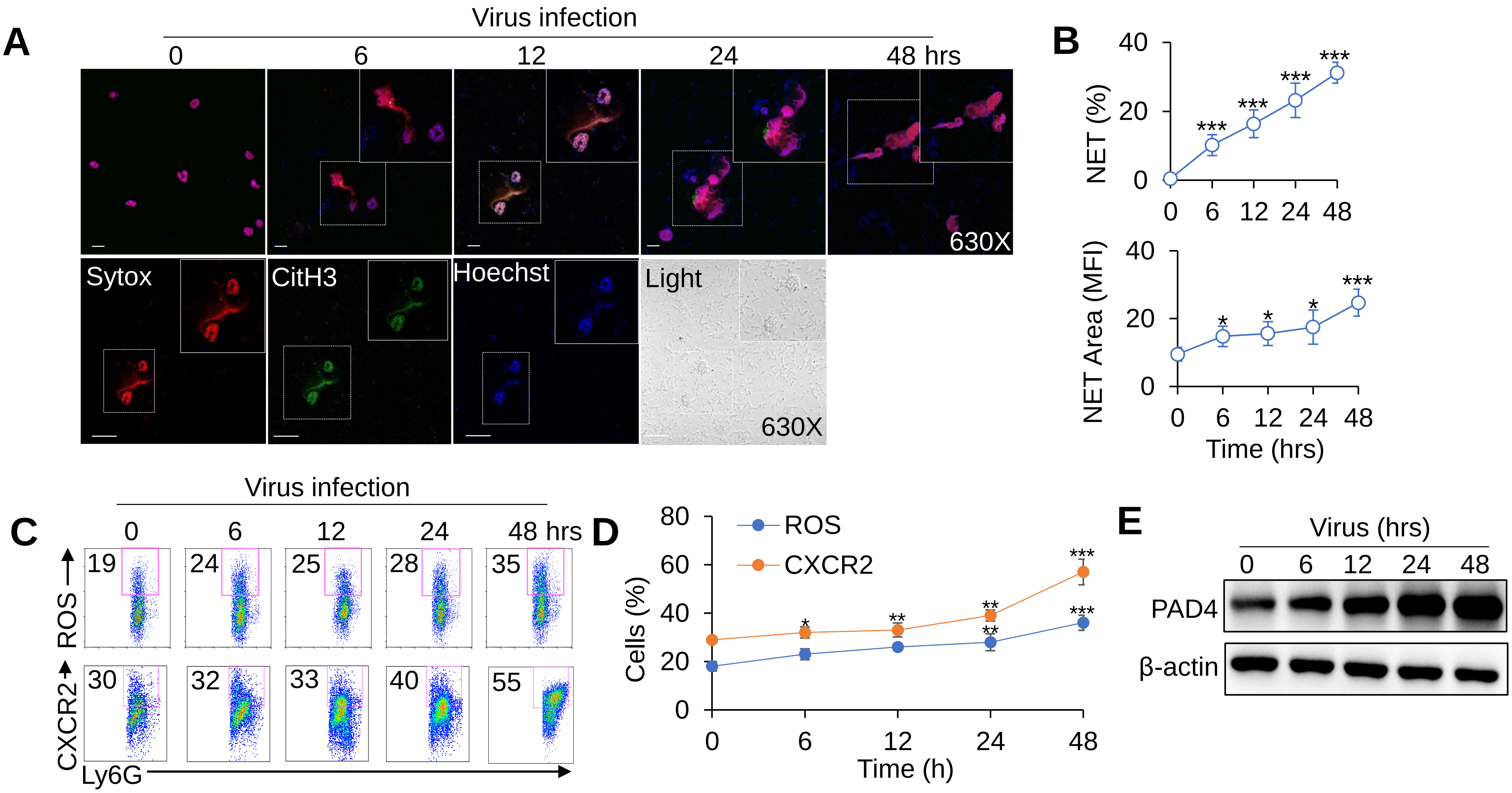

Fig. S2. Neutrophil NETs and macrophage differentiation are related with pulmonary respiratory virus infection. C57BL/6 mice challenged by PR8 virus infection at different time. (**A-B**) NETs of sorted neutrophils from BALF by confocal fluorescence microscope. Typical NET images are displayed (A), and the percent and area of NETs is quantified (B). Scales bars, 50 µm (upper) and 100 µm (lower), original magnification, 630X. (**C-D**) Expression of ROS and CXCR2 in $CD11b^{+}Ly6G^{+}$ neutrophils from BALF. Dot-plots present the representative data from flow cytometry analysis (C), and statistical results are shown (D). (**E**) Western blot of PAD4 in sorted neutrophils from BALF by flow cytometry. The graph summarizes data from three independent experiments with six mice per group. **P*<0.05, ***P*<0.01 and ****P*<0.001, compared with the indicated groups.

Supp. Fig.3

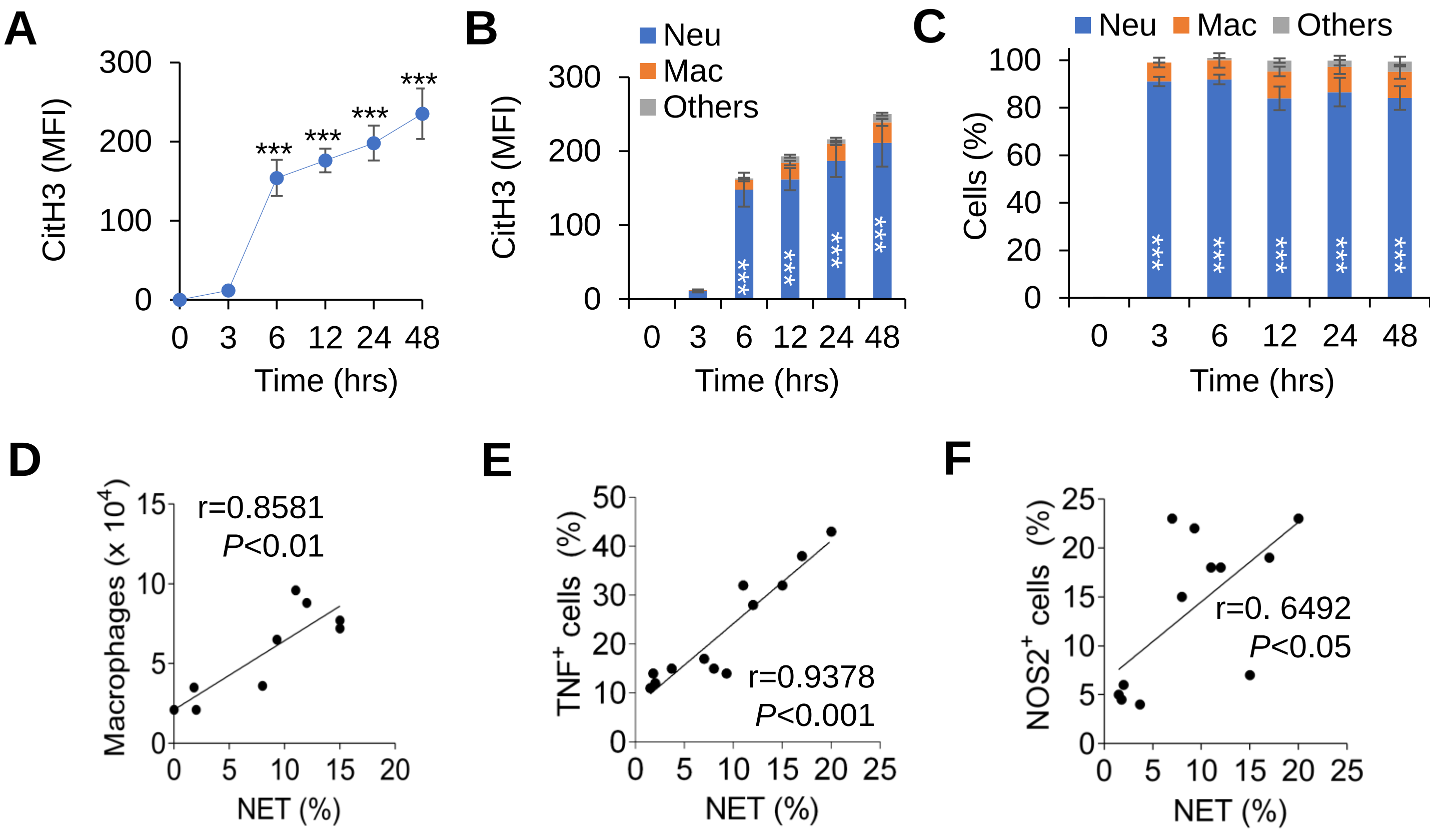

Fig. S3. Neutrophil NETs and macrophage differentiation are related with pulmonary respiratory virus infection.
C57BL/6 mice challenged by PR8 virus infection at different time. (**A-B**) MFI of CitH3 in neutrophils by flow cytometry (A) or in different infiltrating immune cell population (B) from BALF. At each time point, statistical comparison of Neu, Mac, and Other sections. (**C**) NETs of infiltrating immune cell population in BALF in infected mice by confocal fluorescence microscope and analyzed by Image J software. At each time point, statistical comparison of Neu, Mac, and Other sections and statistical results are shown. (**D-F**) The correlations between the NET percent and macrophage number (D), macrophages TNFα^{+} percent (E) or macrophages NOS2^{+} percent (F) in infected mice. The graph summarizes data from three independent experiments with nine to fourteen mice. ***P<0.001, compared with the indicated groups.

Supp. Fig.4

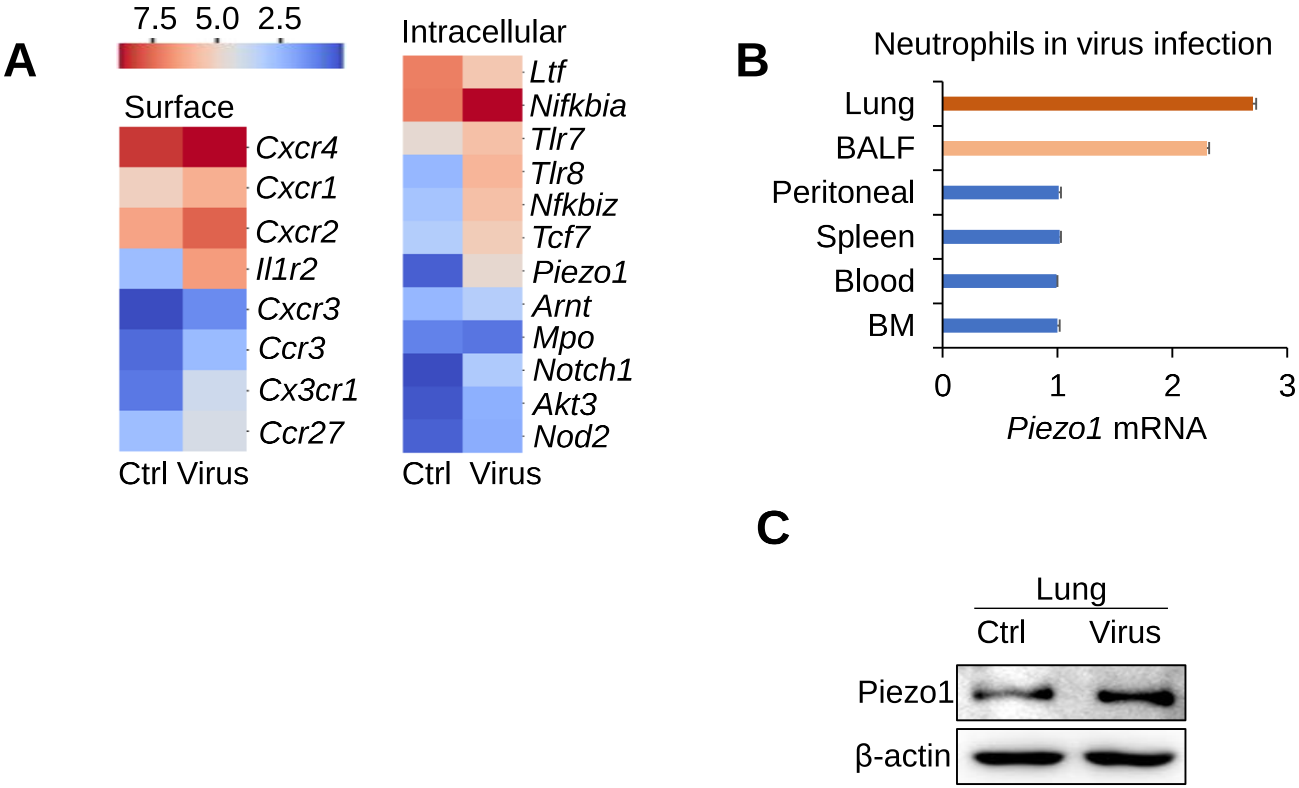

Fig. S4 Piezo1 expressions during virus infection.

(**A**) C57BL/6 mice infected by PR8 virus for 48 hours and lungs were collected. RNA was analyzed by RNA-sequencing to compare the expression profiles of the control and virus-infected cells from lung with certain genes involved in surface and intracellular signaling pathways. (**B**) mRNA expression of *Piezo1* in neutrophils from indicated organ in PR8 virus infected mice. (**C**) Western blot of Piezo1 in neutrophils from lung in virus infected mice as indicated treatment.

Supp. Fig.5

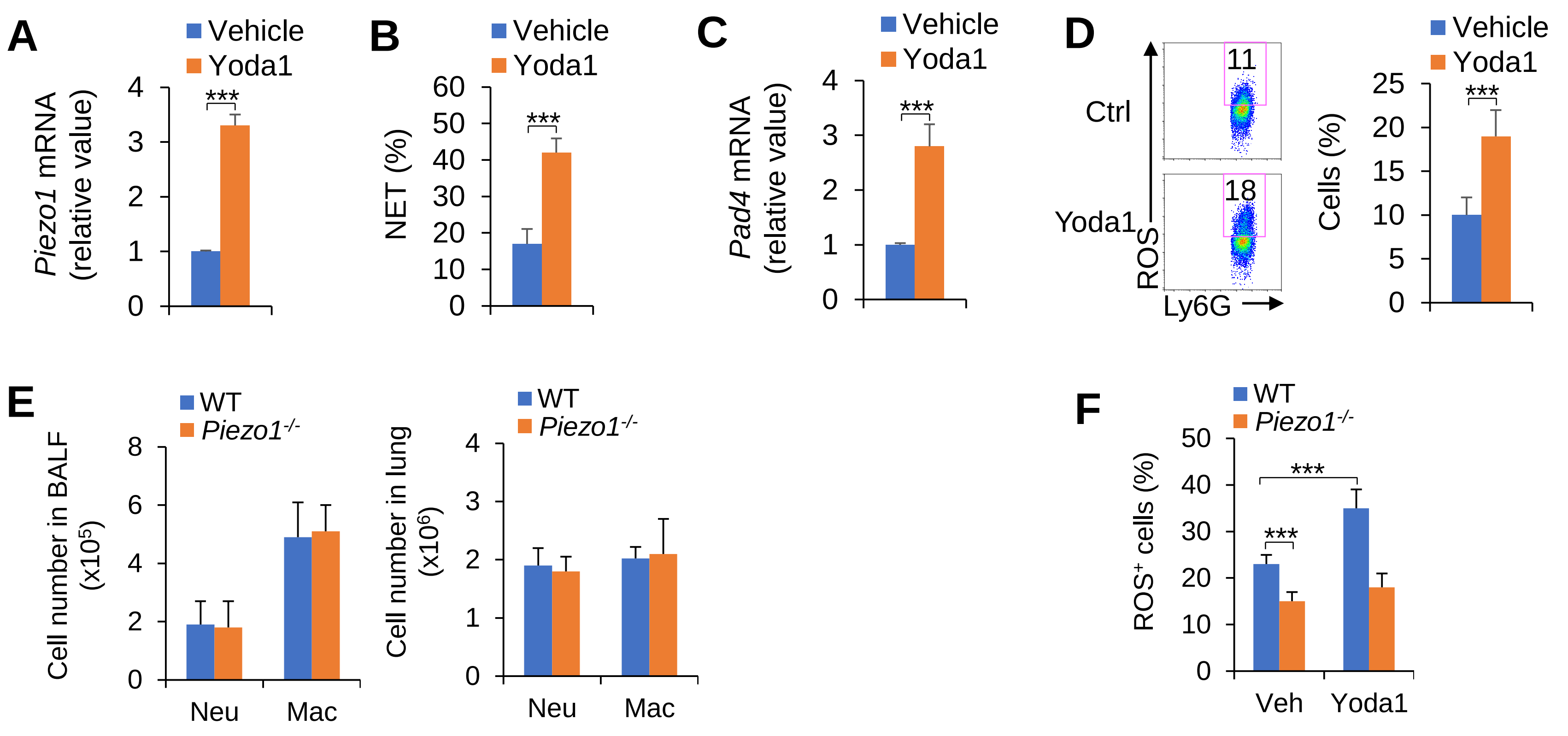

Fig. S5. Piezo1 is sufficient for neutrophils functional activities during virus infection.
Neutrophils isolated from mouse spleen and stimulated by PR8 virus *in vitro* for 6 hours in the presence or absence of Yoda1 (25 μM, MCE). (**A**) *Piezo1* mRNA expressions of neutrophils by qPCR. (**B**) NETs of neutrophils by confocal fluorescence microscope. The percent of NETs is quantified. (**C**) *Pad4* mRNA expressions of neutrophils by qPCR. (**D**) Intracellular staining of ROS in neutrophils by flow cytometry. Dot-plots present the representative data from flow cytometry analysis (left) and statistical results are shown (right). (**E**) Wild-type (WT) mice infected by PR8 virus for 48 hours. Absolute number of $CD11b^{+}Ly6G^{+}$ neutrophils and $CD11b^{+}F4/80^{+}$ macrophages in BALF (left) and lung (right) from infected mice by flow cytometry. (**F**) WT and *Piezo1*$^{-/-}$ mice infected by PR8 virus for 48 hours and treated with or without Yoda1 (2.6 mg/kg, MCE). Expression of ROS in neutrophils isolated from BALF by flow cytometry. Data was summarized. The graph summarizes data from three independent experiments with three mice per group. ***P <0.001, compared with the indicated groups.

Supp. Fig.6

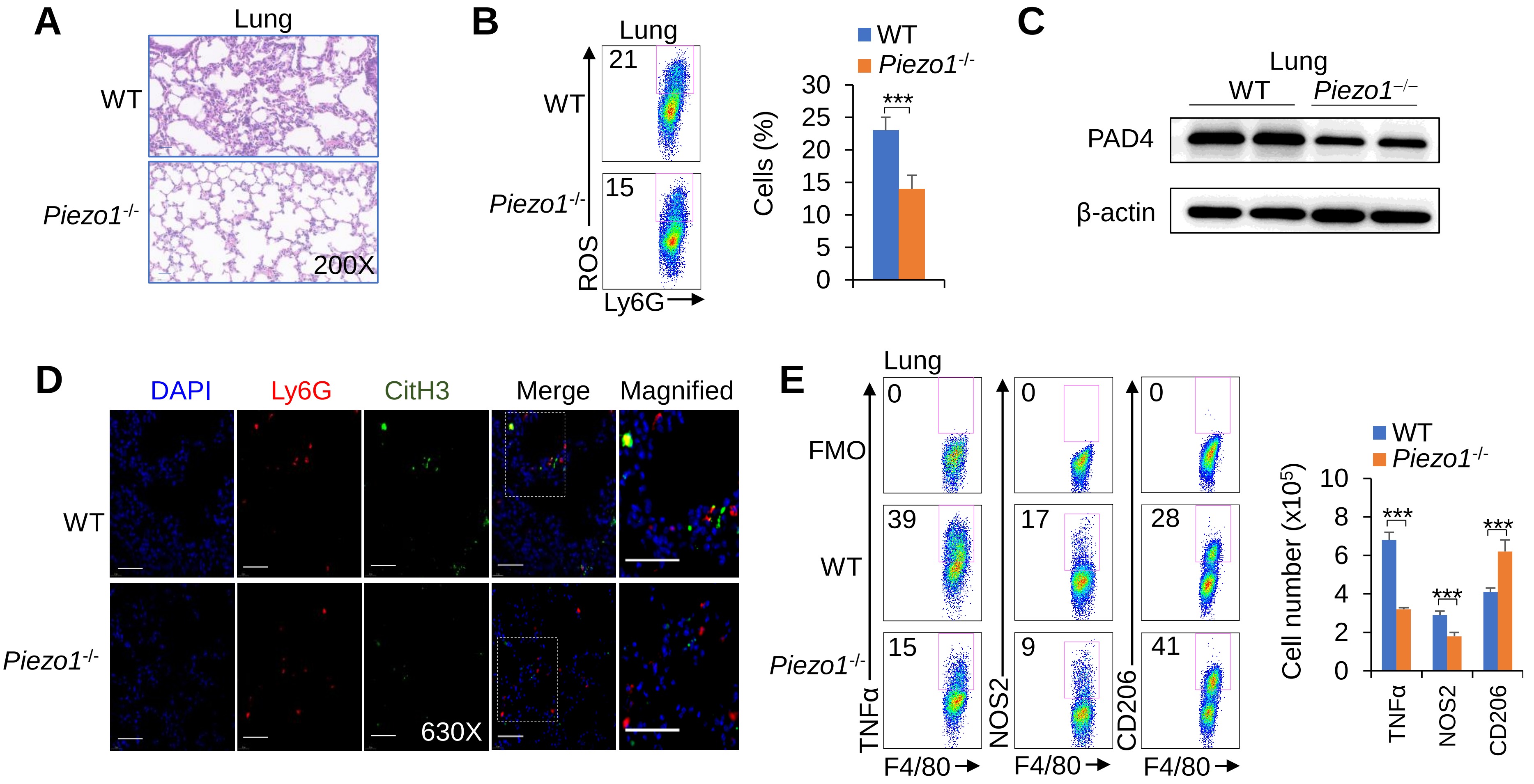

Fig. S6. Piezo1 regulates the neutrophil NET formation and macrophage differentiation during virus infection. Wild-type (WT) and *Piezo1*$^{-/-}$ mice infected by PR8 virus for 48 hours. (**A**) Hematoxylin and eosin (H&E) staining of infected mouse lungs. Scales bars, 20 µm, original magnification, 200X. (**B**) Expression of ROS in neutrophils isolated from lung by flow cytometry. Dot-plots present the representative data (left) and data summarized (right). (**C**) Western blot of PAD4 in neutrophils isolated from lung. (**D**) NETs of neutrophils in lung by confocal fluorescence microscope. Typical NET images are displayed. Scales bars, 50 µm, original magnification, 630X. (**E**) Intracellular staining of TNFα and NOS2 and expression of CD206 in CD11b^{+}F4/80^{+} macrophages isolated from lung from virus-infected mice by flow cytometry. Fluorescence Minus One control, FMO. The graph summarizes data from three independent experiments with four mice per group. ***P<0.001, compared with the indicated groups.

Supp. Fig.7

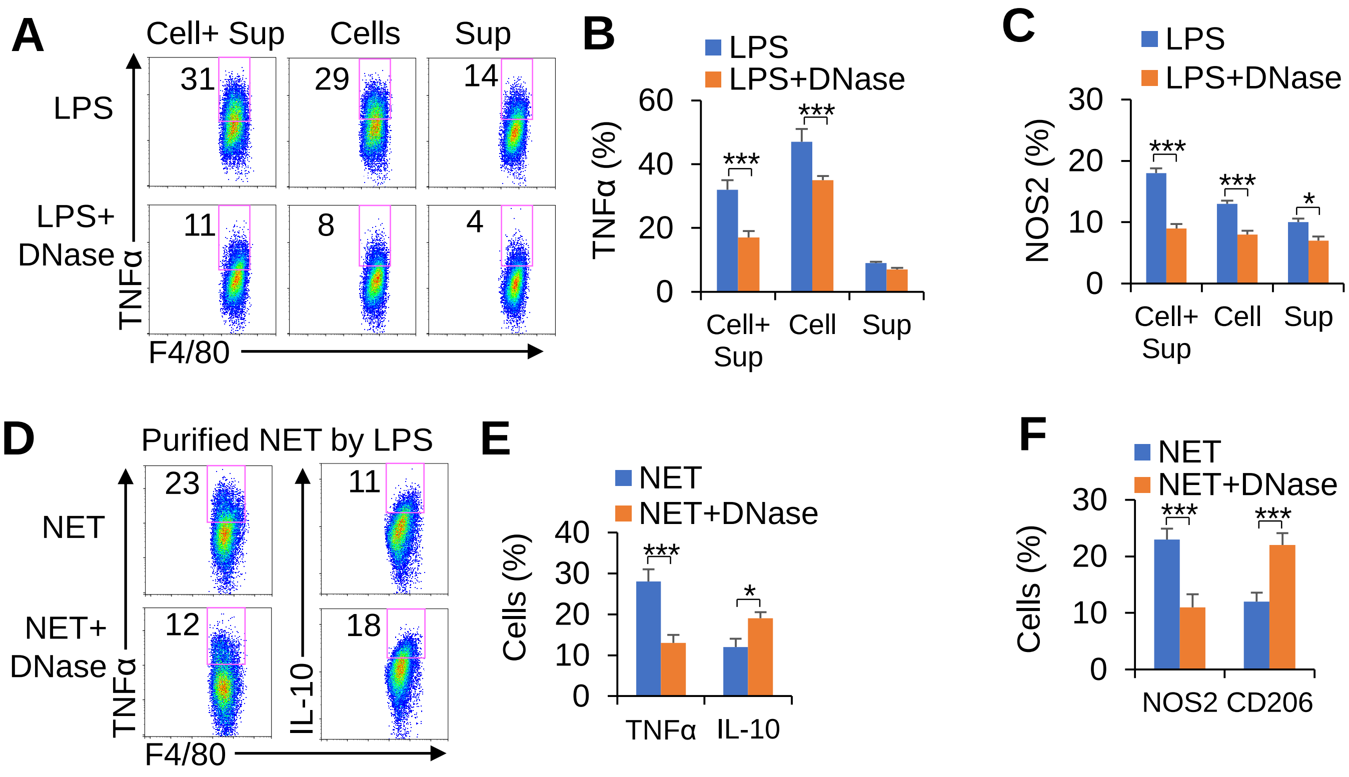

Fig. S7. Neutrophils NET directed M1 macrophage differentiation during virus infection.

(**A-C**) Neutrophils isolated from mouse spleen and stimulated by LPS (10 ng/ml) *in vitro* for 6 hours and cells or/and cell culture supernatant were collected for the subsequent experiment. Bone marrow-derived macrophages (BMDM) were treated by neutrophils culture collects as indicated and stimulated by LPS or LPS + DNase I for 6 hours. Intracellular staining of TNFα (A-B) and NOS2 (C) in macrophages was determined by flow cytometry. Dot-plots present the representative data from flow cytometry analysis (A), and statistical results are shown (B&C). (**D-F**) Sorted neutrophils isolated from mouse spleen and stimulated by LPS *in vitro* for 6 hours and NET DNA was purified and collected for the subsequent experiment. BMDM were treated by NET DNA (1 ng/μl) from neutrophils stimulated by LPS for 6 hours. Intracellular staining of TNFα and IL-10 (D-E), NOS2 and CD206 (F) in macrophages was determined by flow cytometry. Dot-plots present the representative data from flow cytometry analysis (D), and statistical results are shown (E&F). The graph summarizes data from three to four independent experiments with four mice per group. *P<0.05 and ***P<0.001, compared with the indicated groups.

Supp. Fig.8

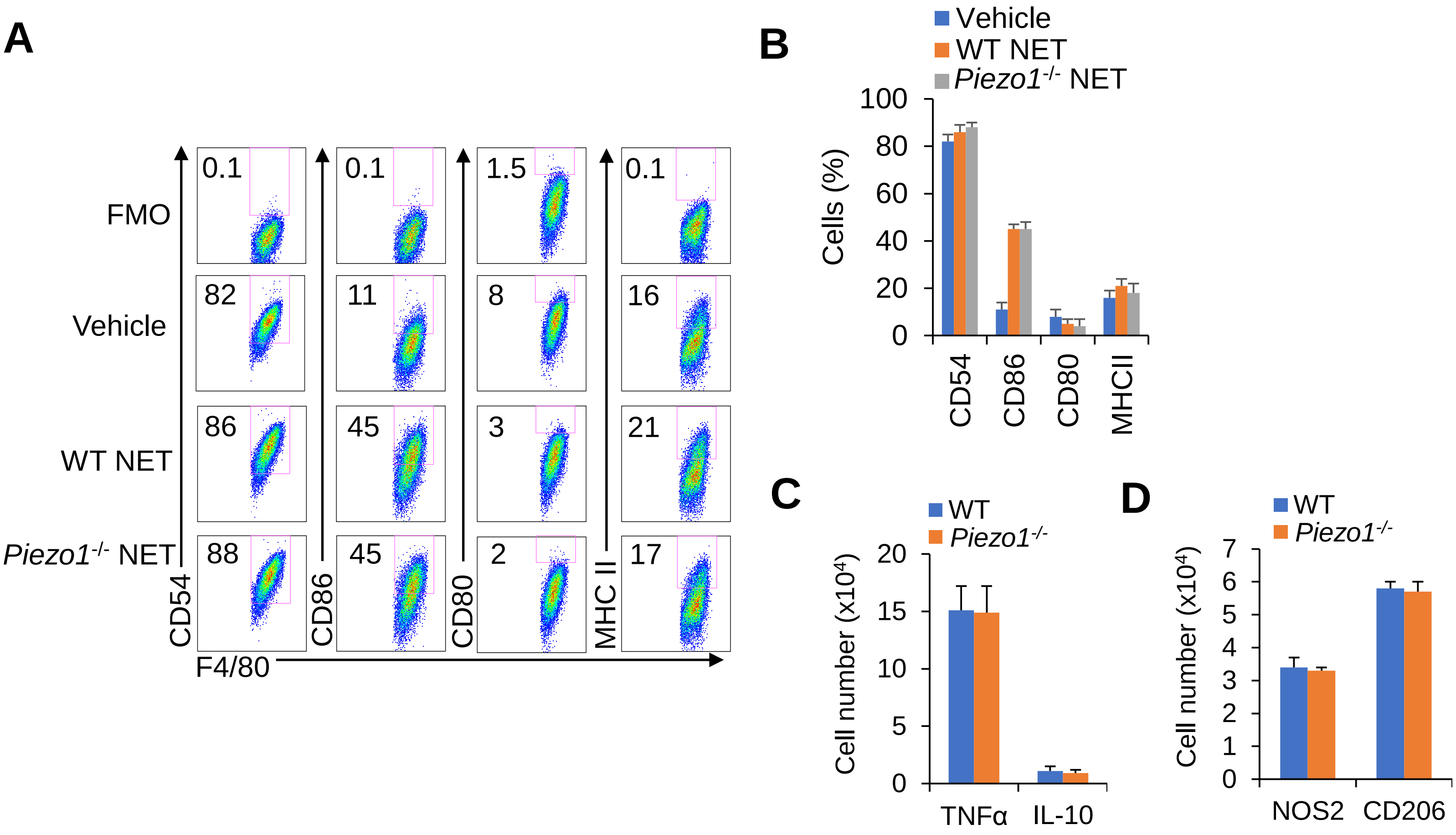

Fig. S8. Neutrophil NET DNA effects on M1 macrophage differentiation.
(**A-B**) Sorted neutrophils isolated from wild-type (WT) and *Piezo1*$^{-/-}$ mouse spleen and stimulated by LPS *in vitro* for 6 hours and NET DNA was purified and collected for the subsequent experiment. Bone marrow-derived macrophages (BMDM) were treated by NET DNA (1 ng/µl) from the same number neutrophils of WT and *Piezo1*$^{-/-}$ mice for 6 hours. Expression of indicated molecules in macrophages. Dot-plots present the representative data from flow cytometry analysis (A), and statistical results are shown (B). Fluorescence Minus One control, FMO. (**C-D**) WT and *Piezo1*$^{-/-}$ BMDMs were stimulated by virus for 6 hours. Intracellular staining of TNFα and IL-10, NOS2 and expressions of CD206 in macrophages was determined by flow cytometry. Statistical results are shown. The graph summarizes data from three independent experiments with four mice per group.

Supp. Fig.9

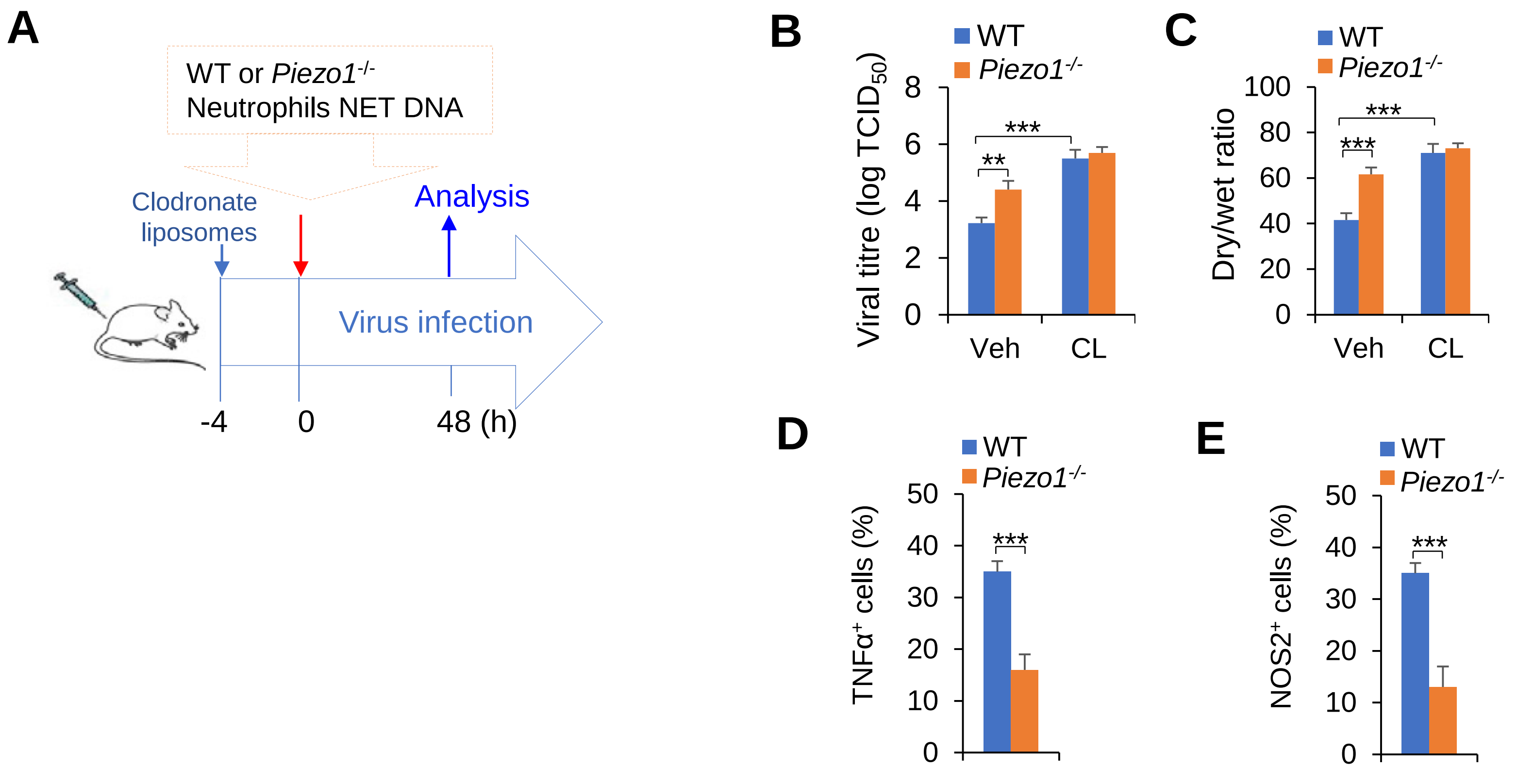

Fig. S9. *Piezo1*$^{-/-}$ neutrophil NET directed M1 macrophage differentiation during virus infection.
Neutrophils isolated from WT or *Piezo1*$^{-/-}$ mouse spleen and stimulated by PR8 virus *in vitro* for 6 hours and NET DNA was purified and collected for the subsequent experiment. NET DNA from the neutrophils (1 x 10^7) from WT or *Piezo1*$^{-/-}$ mice was i.v. injected into recipient mice at 0 hours. Simultaneously, recipient mice were pretreated by clodronate liposomes (CL) i.p. injection (100 µl) for macrophage depletion at -4 hours and 24 hours. At 0 hour, mice were challenging with PR8 virus for 48 hours (**A**). (**B**) Lung virus titre of infected mice. $TCID_{50}$, data are shown in log_{10} scale per lung lobe. (**C**) Ratio of dry to wet weight of lungs from the infected mice at 48 hours. Intracellular staining of TNFα (**D**) and NOS2 (**E**) in macrophages in BALF from the indicated mice. The graph summarizes data from three independent experiments with three or four mice per group. ***P*<0.01 and ****P*<0.001, compared with the indicated groups.

Supp. Fig.10

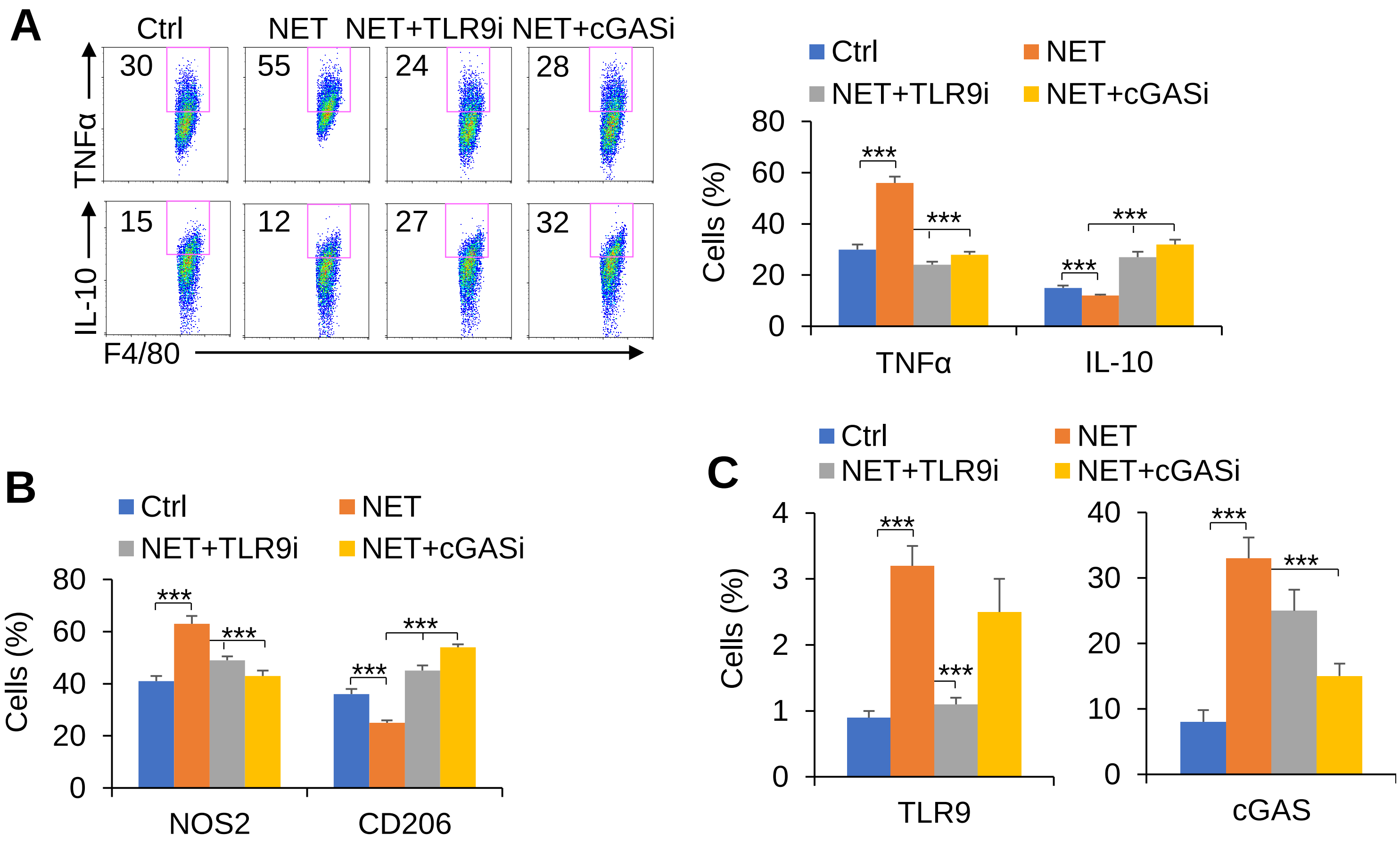

Fig. S10. Neutrophil NET regulates M1 macrophage differentiation through TLR9-cGAS signaling. Neutrophils isolated from mouse spleen and stimulated by virus *in vitro* for 6 hours and NET DNA was purified and collected for the subsequent experiment. Bone marrow-derived macrophages were treated by NET DNA (1 ng/μl) from neutrophils stimulated by LPS with or without TLR9 inhibitor (E6446 dihydrochloride, 1 μM, Selleck, USA) or cGAS inhibitor (RU.521, 10 μM, Selleck, USA) for 6 hours. (**A**) Intracellular staining of TNFα and IL-10 in macrophages by flow cytometry. Dot-plots present the representative data from flow cytometry analysis (left), and statistical results are shown (right). (**B**) Intracellular staining of NOS2 and CD206 in macrophages by flow cytometry and statistical results are shown. (**C**) Expressions of TLR9 and cGAS in macrophages by flow cytometry and statistical results are shown. The graph summarizes data from three independent experiments with three mice per group. ****P* <0.001, compared with the indicated groups.

Supp. Fig.11

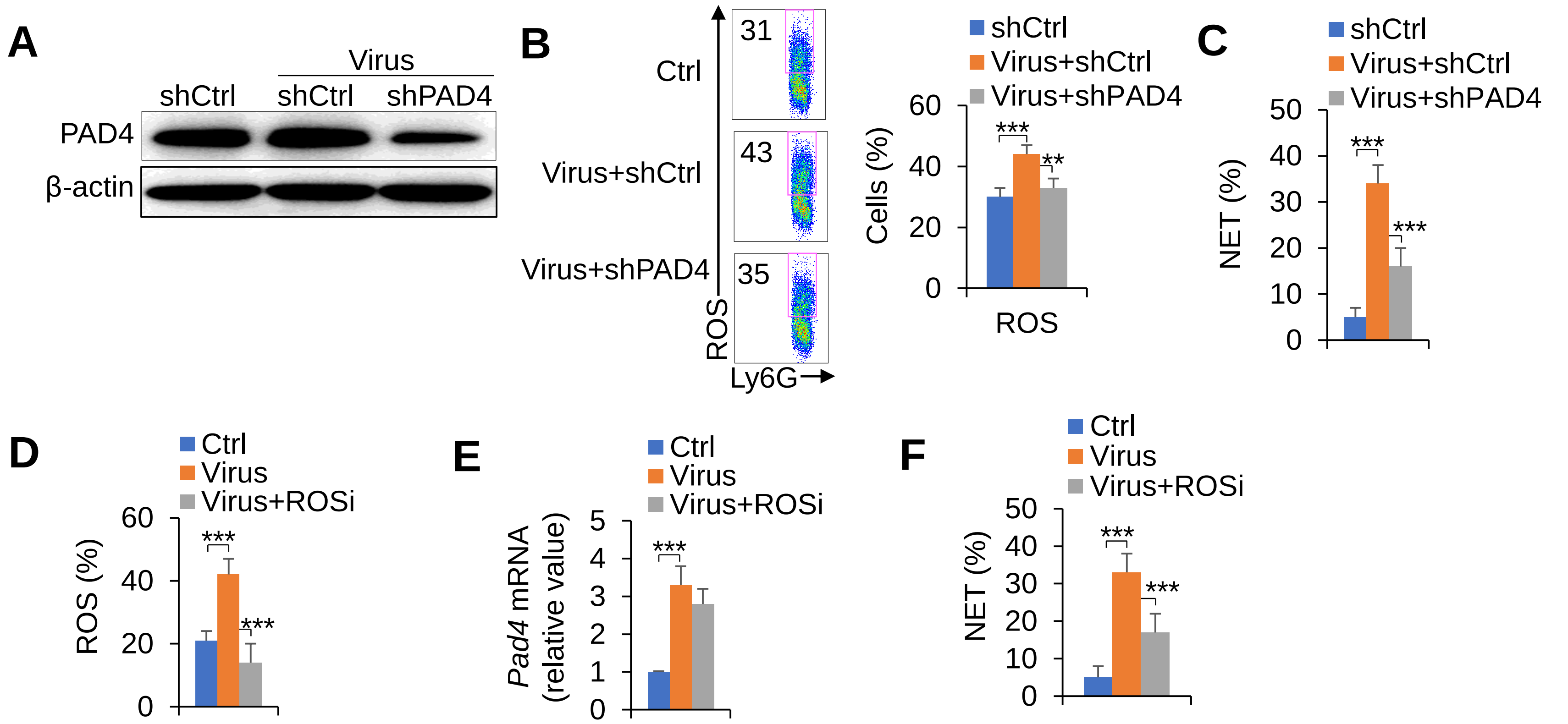

Fig. S11. ROS is required for PAD4-dependent neutrophil NET formation during virus infection.
(**A-C**) Neutrophils isolated from mouse spleen and transfected with shRNA control (ctrl) or shRNA PAD4 (shPAD4). Neutrophils stimulated by virus for 6 hours. (A) Western blot of PAD4 in neutrophils. (B) Intracellular staining of ROS in neutrophils. Dot-plots present the representative data from flow cytometry analysis (left), and statistical results are shown (right). (C) NETs of neutrophils by confocal fluorescence microscope. The percent of NETs is quantified. (**D-F**) C57BL/6 mice challenged by PR8 virus with or without ROS inhibitor (HTHQ, 5 μM, MCE) treatment. (D) Expression of ROS in CD11b^{+}Ly6G^{+} neutrophils from BALF by flow cytometry and statistical results are shown. (E) *Pad4* mRNA expression in sorted the CD11b^{+}Ly6G^{+} neutrophils from BALF. (F) NETs of neutrophils by confocal fluorescence microscope. The percent of NETs is quantified. The graph summarizes data from three independent experiments with three samples per group. ***P*<0.01 and ****P*<0.001, compared with the indicated groups.

Supp. Fig.12

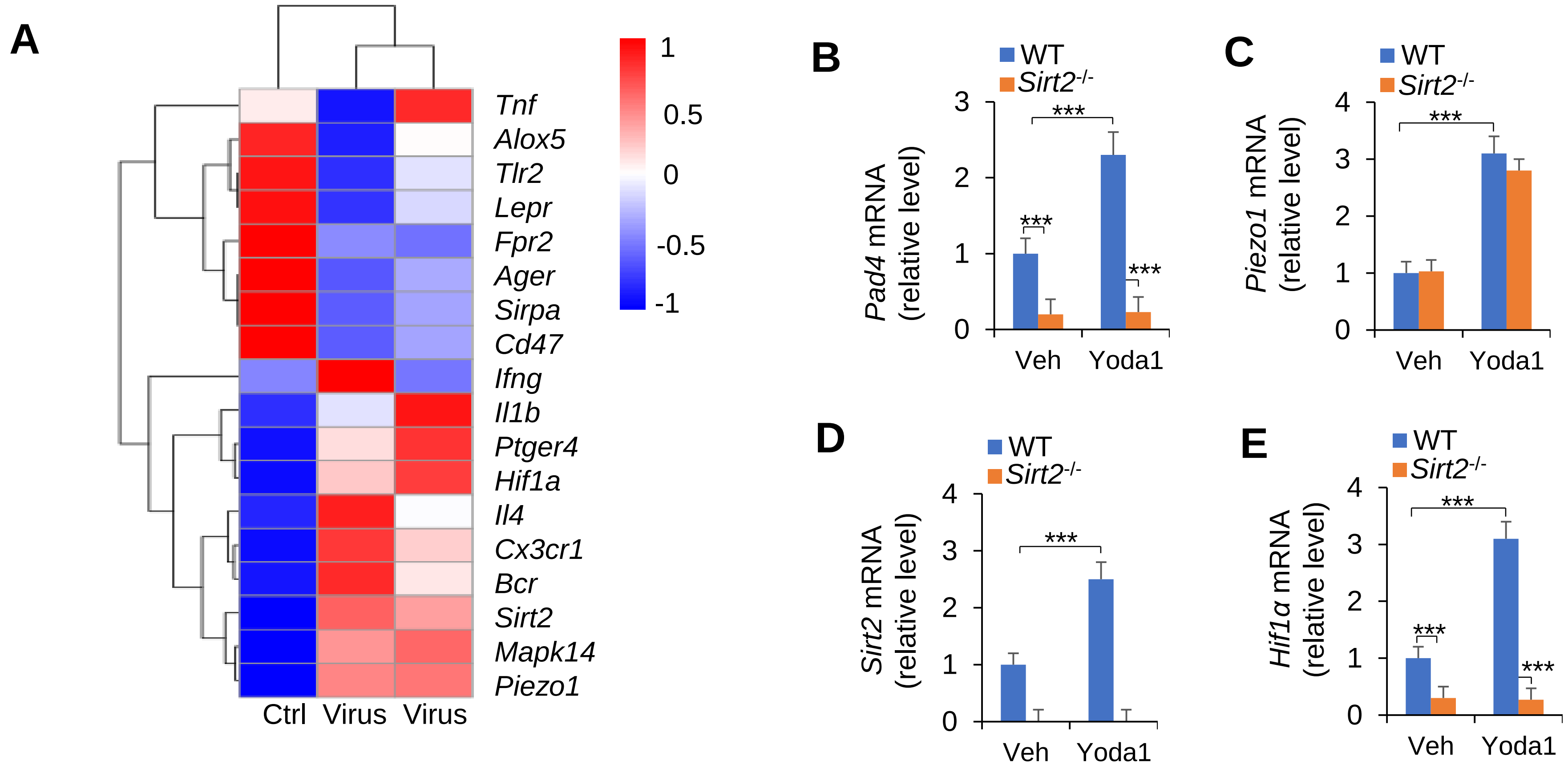

Fig. S12. Piezo1 directs neutrophil NET formation though SIRT2-HIF1α signaling during virus infection.
(**A**) C57BL/6 mice infected by PR8 virus for 48 hours and lungs were collected. RNA was analyzed by RNA sequencing to compare the expression profiles of the control and virus-infected cells from lung with certain genes involved in ROS, cytokine and chemokine signaling pathways. (**B-E**) *Pad4, Piezo1, Sirt2 and Hif1α* mRNA expression in sorted neutrophils from BALF from virus infected WT and *Sirt2*$^{-/-}$ mice treatment with or without Yoda1 (2.6 mg/kg, MCE). The graph summarizes data from three independent experiments with four mice per group. ****P*<0.001, compared with the indicated groups.

Supp. Fig.13

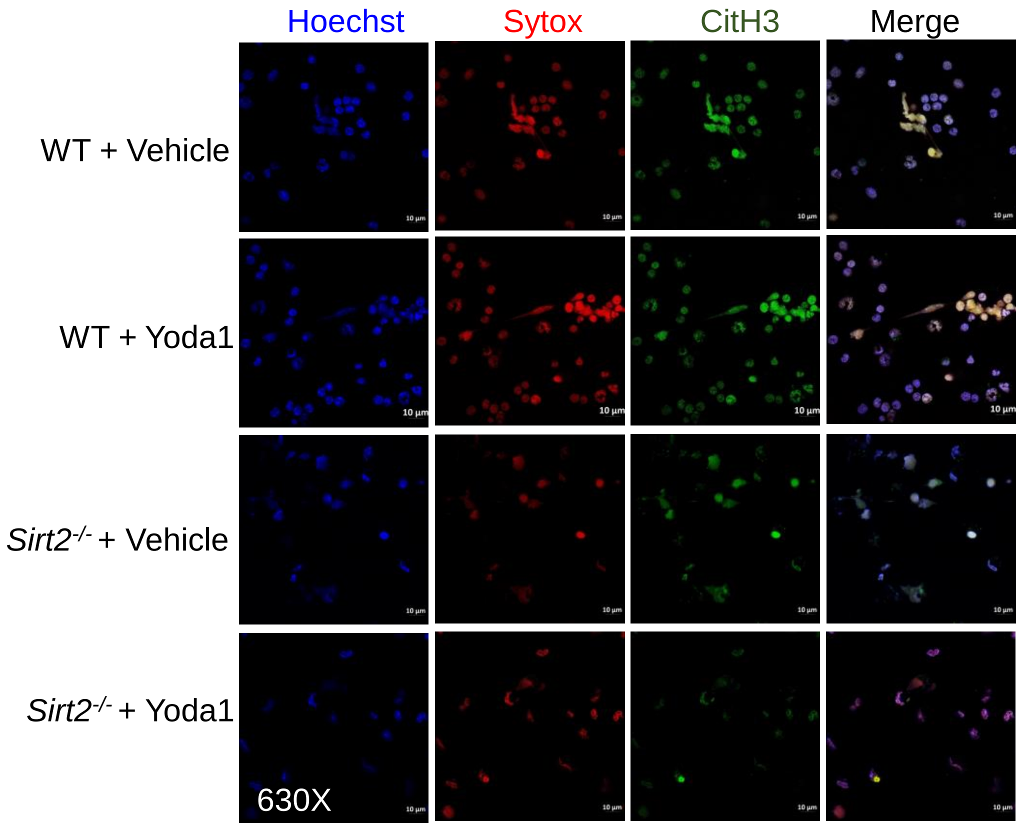

Fig. S13. Piezo1-SIRT2 regulates the neutrophil NET formation during virus infection.
Wild-type (WT) and *Sirt2*$^{-/-}$ mice infected by PR8 virus for 48 hours and treated with or without Yoda1 (2.6 mg/kg, MCE). NETs of neutrophils isolated from BALF by confocal fluorescence microscope. Typical NET images are shown. Scales bars, 10 µm, original magnification, 630X.

Supp. Fig.14

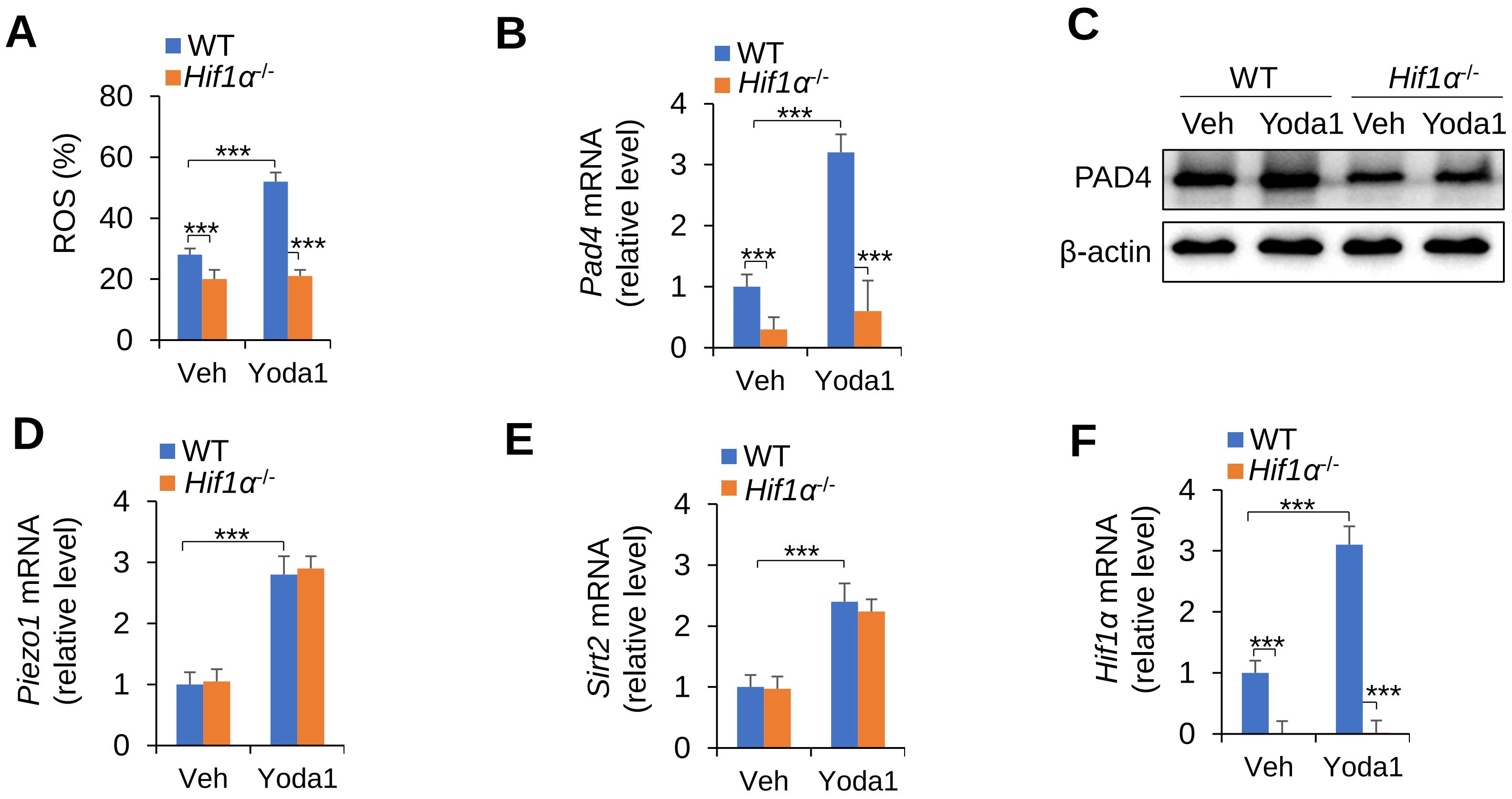

Fig. S14. Piezo1-HIF1α directs neutrophil NET formation during virus infection.
(**A**) Wild-type (WT) and *Hif1α*$^{-/-}$ mice infected by PR8 virus for 48 hours and treatment with or without Yoda1 (2.6 mg/kg). Expression of ROS in neutrophils isolated from BALF by flow cytometry and data summarized. (**B-C**) *Pad4* mRNA (B) and protein (C) expression in sorted neutrophils from BALF from virus infected WT and *Hif1α*$^{-/-}$ mice treatment with or without Yoda1 (2.6 mg/kg, MCE). (**D-F**) *Piezo1, Sirt2 and Hif1α* mRNA expression in sorted neutrophils from BALF from virus infected WT and *Hif1α*$^{-/-}$ mice treatment with or without Yoda1 (2.6 mg/kg, MCE). The graph summarizes data from three independent experiments with four mice per group. ***P <0.001, compared with the indicated groups.

Supp. Fig.15

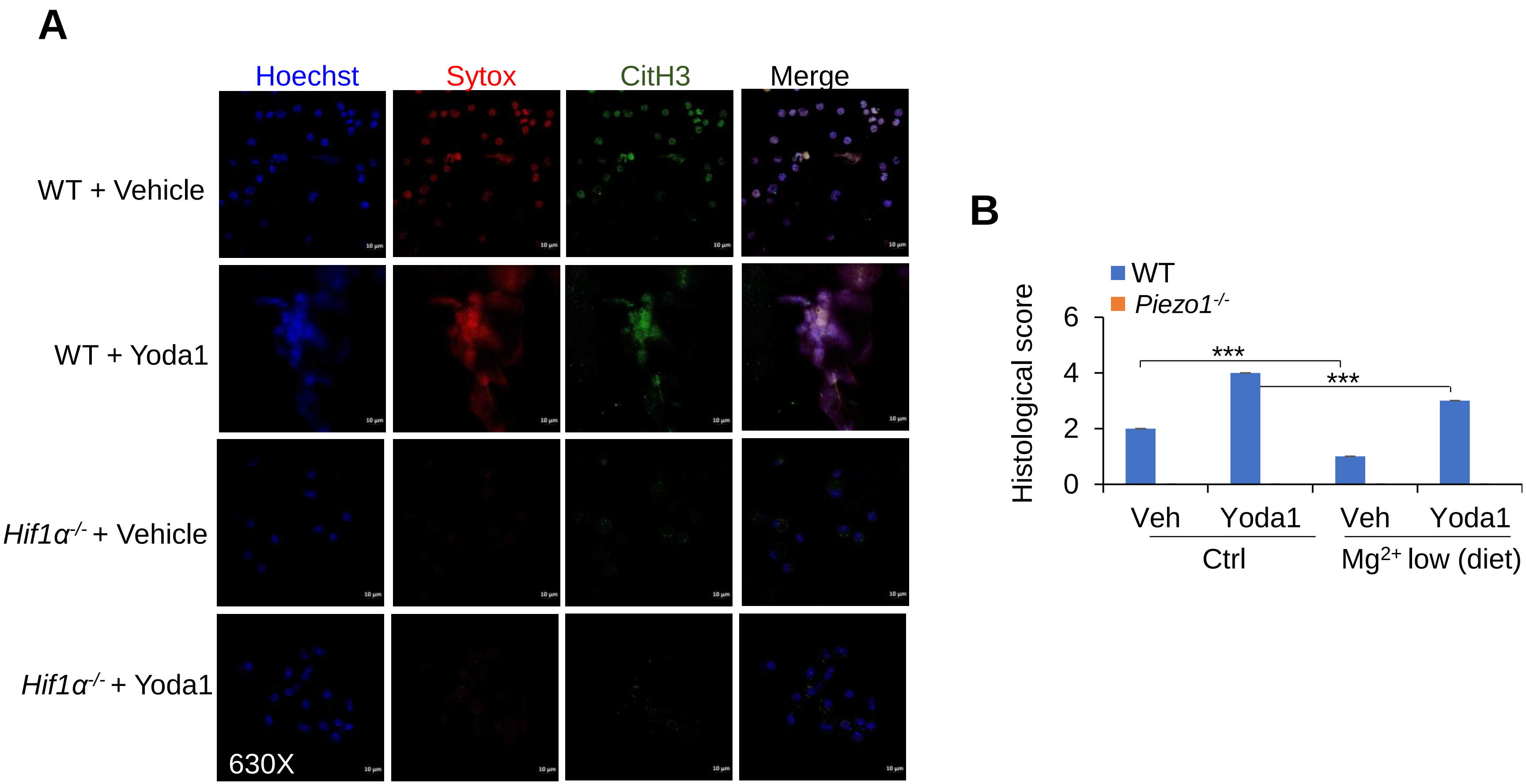

Fig. S15. Piezo1 regulates the neutrophil NET formation and histological inflammation during virus infection.
(**A**) Wild-type (WT) and *Hif1α*$^{-/-}$ mice infected by PR8 virus for 48 hours and treated with or without Yoda1 (2.6 mg/kg, MCE). NETs of neutrophils isolated from BALF by confocal fluorescence microscope. Typical NET images are displayed. Scales bars, 10 μm, original magnification, 630X. (**B**) WT or *Piezo1*$^{-/-}$ mice infected by PR8 virus for 48 hours and treated with or without Yoda1 (2.6 mg/kg, MCE) under normal or low magnesium diet condition. Inflammatory cell infiltration score in infected mouse lung tissue with hematoxylin and eosin (H&E) staining. The graph summarizes data from three independent experiments with three mice per group. ***P<0.001, compared with the indicated groups.

Supp. Fig.16

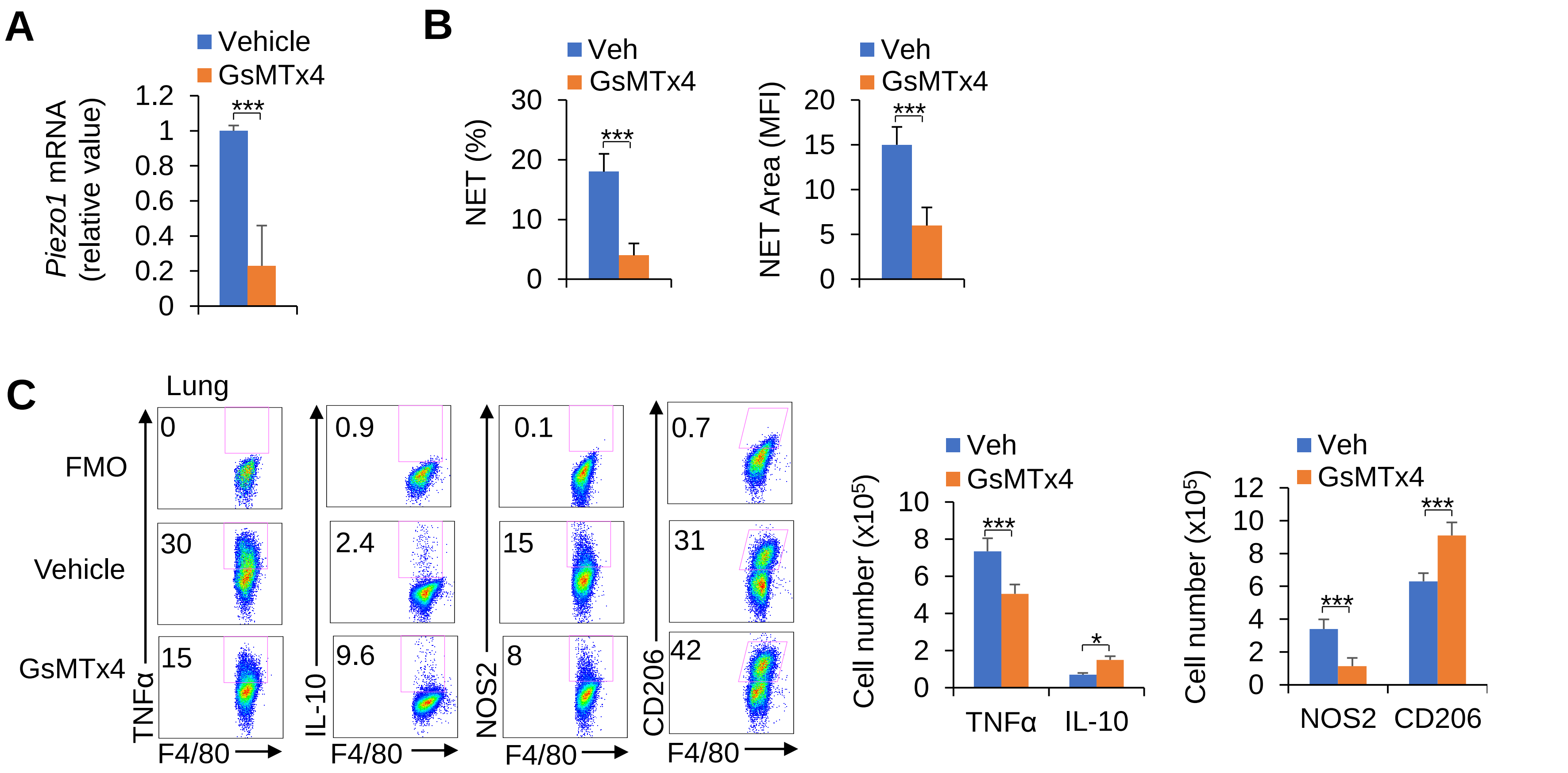

Fig. S16. Piezo1 is necessary for the neutrophil NET formation and macrophage differentiation during virus infection. Wild-type (WT) infected by PR8 virus for 48 hours and treated with or without GsMTx4 (2.0 mg/kg, MCE). (**A**) *Piezo1* mRNA expressions of neutrophils by qPCR. (**B**) NETs of neutrophils isolated from BALF by confocal fluorescence microscope. The percent and area of NETs is quantified. (**C**) Intracellular staining of TNFα, IL-10, NOS2, and expression of CD206 in $CD11b^+F4/80^+$ macrophages isolated from lung from virus infected mice by flow cytometry. Dot-plots present the representative data from flow cytometry analysis (left), and statistical results are shown (right). Fluorescence Minus One control, FMO. The graph summarizes data from three independent experiments with four mice per group. ***P<0.001, compared with the indicated groups.

Supp. Fig.17

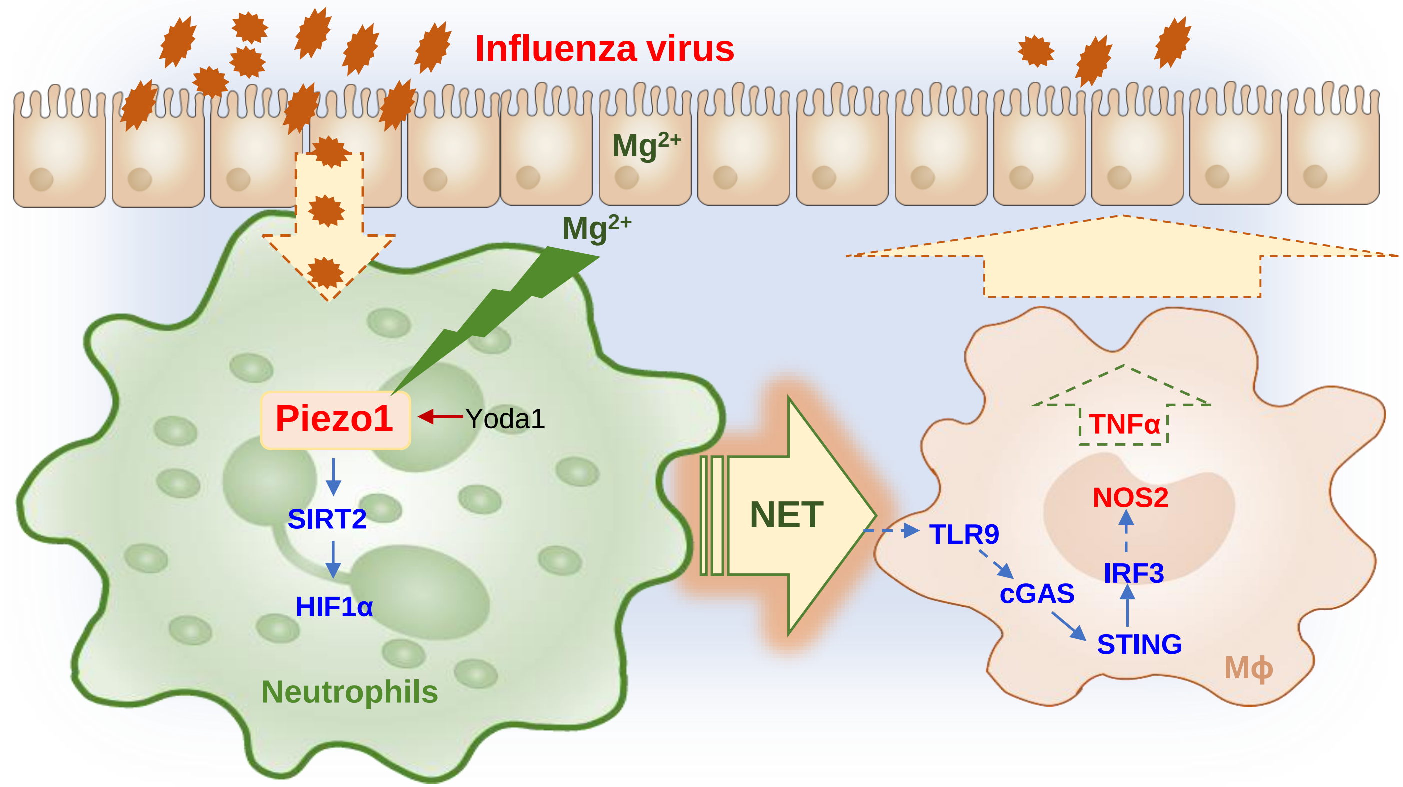

Fig.S17. Magnesium sensing Pizeo1-directed neutrophils extracellular trap regulates macrophage differentiation during influenza virus infection.
Proposed model of how Piezo1 in neutrophils responses to influenza virus signals to trigger NET formation and regulate the M1 macrophage differentiation in anti-virus immunity.

Supp. Fig.18

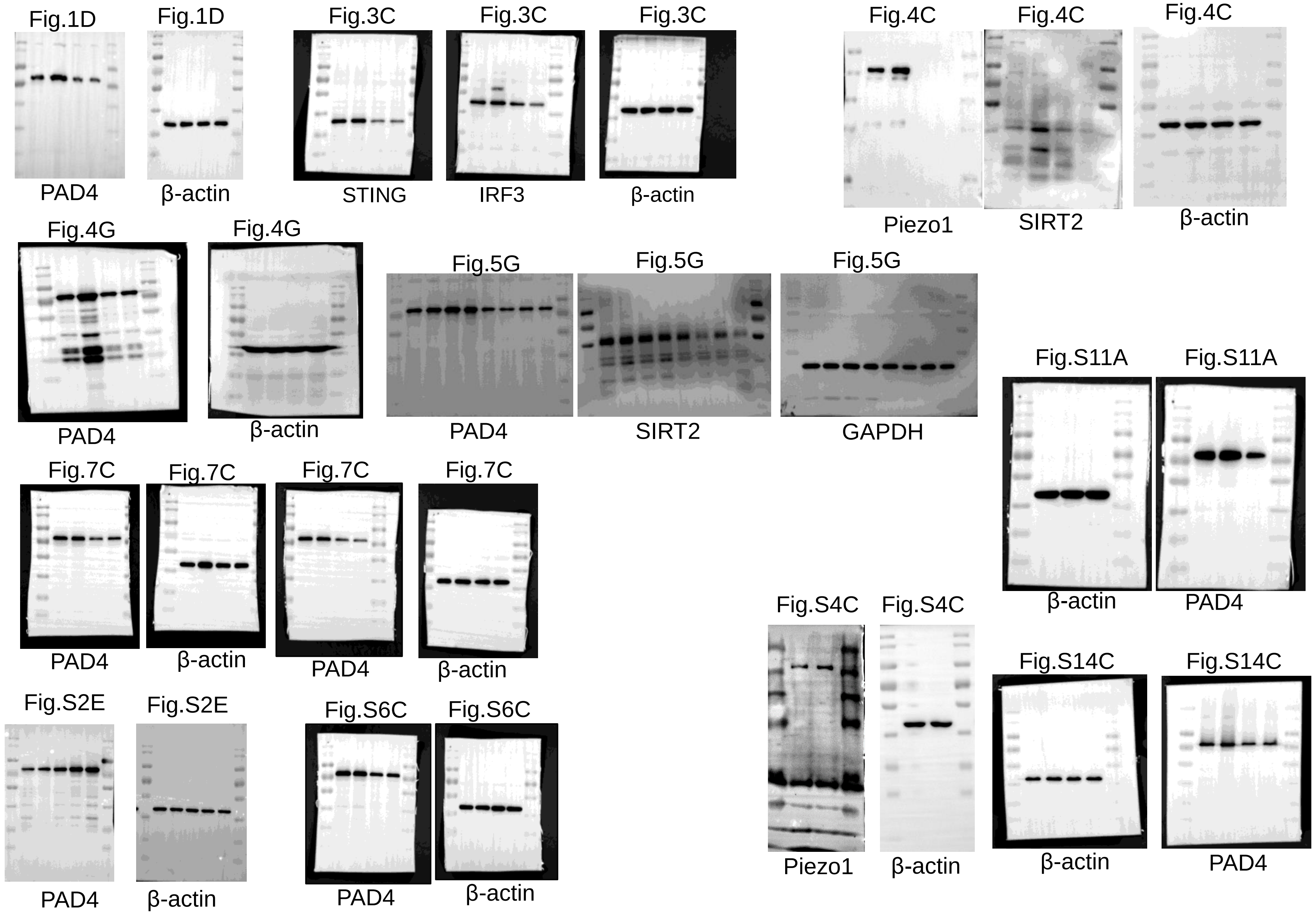

Fig.S18. Western blot of uncroped images of whole membranes.
