# Supplementary material for: Piezo1-directed neutrophil extracellular traps regulate macrophage differentiation during influenza virus infection
Source: Cell Death Dis. 2025 Jan 31;16(1):60. doi: 10.1038/s41419-025-07395-5 (PMC11785962; doi:10.1038/s41419-025-07395-5)

Original data of qPCR

Fig.3B

Exp1		Tlr9	cGas	Sting	Irf3
	WT	1	1	1	1
	Piezo1-/-	0.5209552	0.435104734	0.5674416	0.5361045
		Tlr9	cGas	Sting	Irf3
Exp2	WT	1	1	1	1
	Piezo1-/-	0.6399445	0.524167125	0.64246214	0.6375395
Exp3		Tlr9	cGas	Sting	Irf3
	WT	1	1	1	1
	Piezo1-/-	0.4207694	0.37329522	0.54424074	0.4629273
Average		Tlr9	cGas	Sting	Irf3
	WT	1	1	1	1
	Piezo1-/-	0.527223	0.444189026	0.58471483	0.5455238
Sd		Tlr9	cGas	Sting	Irf3
	WT	0	0	0	0
	Piezo1-/-	0.1097219	0.07584508	0.05133843	0.0876863

Fig.4B

Exp1		Cxcr1	Sirt1	Sirt2	Hif1a	Hif1b	Pad4
	WT	1	1	1	1	1	1
	Piezo1-/-	0.828144512	0.781869941	0.4337335	0.4789139	0.93066659	0.345139
Exp2		Cxcr1	Sirt1	Sirt2	Hif1a	Hif1b	Pad4
	WT	1	1	1	1	1	1
	Piezo1-/-	0.945040045	0.862404905	0.4964624	0.5193778	0.96851976	0.355685
Exp3		Cxcr1	Sirt1	Sirt2	Hif1a	Hif1b	Pad4
	WT	1	1	1	1	1	1
	Piezo1-/-	0.749543384	0.790584996	0.3704149	0.43567	0.88340346	0.2597257
Aveage		Cxcr1	Sirt1	Sirt2	Hif1a	Hif1b	Pad4
	WT	1	1	1	1	1	1
	Piezo1-/-	0.840909314	0.811619947	0.4335369	0.4779872	0.92752994	0.3201832
SD		Cxcr1	Sirt1	Sirt2	Hif1a	Hif1b	Pad4
	WT	0	0	0	0	0	0
	Piezo1-/-	0.098371446	0.044196402	0.063024	0.0418616	0.04264476	0.0526226

Fig.5C

PAD4						
		Exp1		Exp2		Exp3
	WT-Veh	1		1		1
	KO-Veh	0.352915		0.380894		0.324916
	WT-Ruth	0.956607		1.046023		0.763918
	KO-Ruth	0.419681		0.340638		0.467699
Aveage				SD		
	WT-Veh	1			WT-Veh	0
	KO-Veh	0.352908			KO-Veh	0.027989
	WT-Ruth	0.922183			WT-Ruth	0.144169
	KO-Ruth	0.409339			KO-Ruth	0.064159

Western blot of uncroped images of whole membranes

Fig.1D

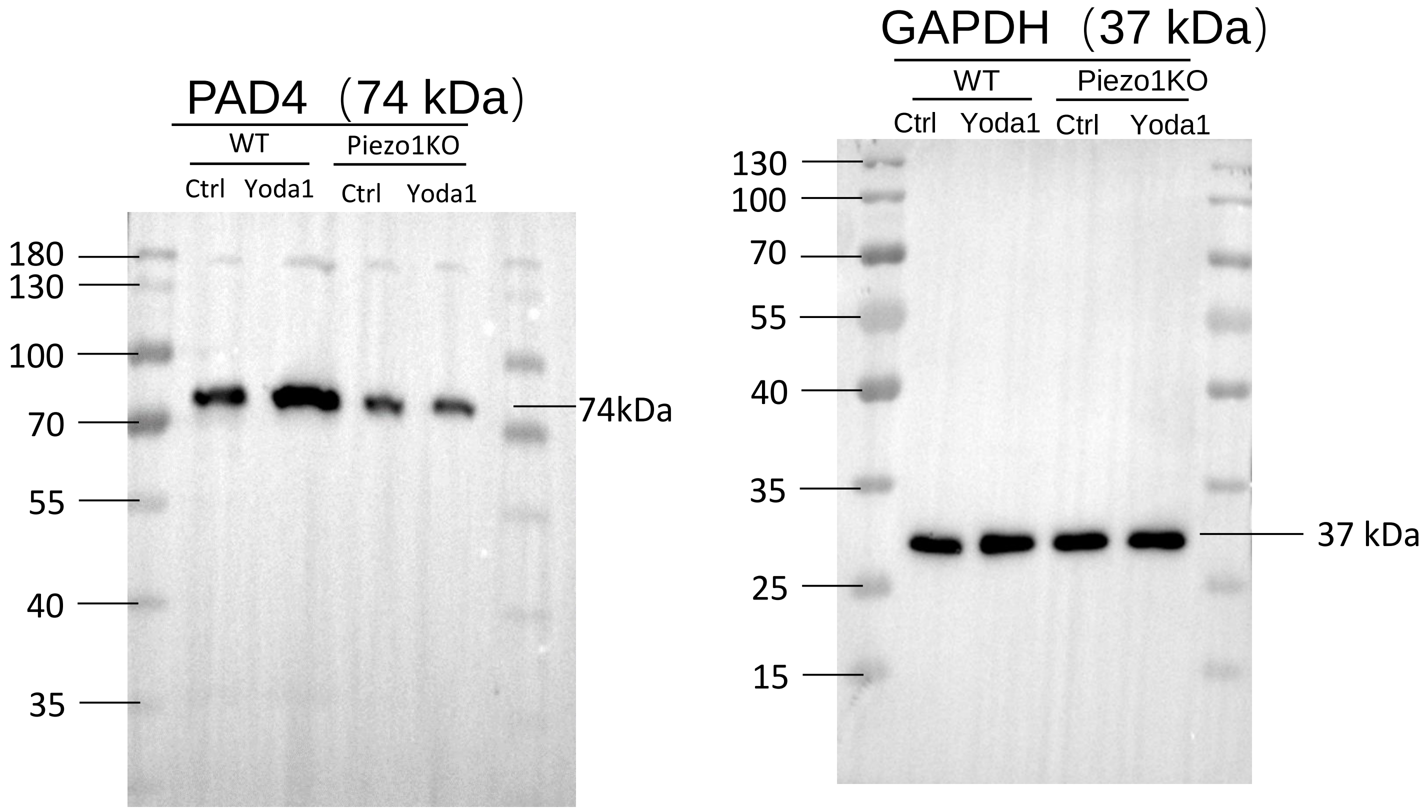

PAD4 (74 kDa)
WT
Piezo1KO
Ctrl
Yoda1
Ctrl
Yoda1
180
130
100
70
55
40
35
74kDa
GAPDH (37 kDa)
WT
Piezo1KO
Ctrl
Yoda1
Ctrl
Yoda1
130
100
70
55
40
35
25
15
37 kDa

Fig.3C

Western blot
Organ: lung

Sting (40 kDa) IRF3 (50 kDa) β-actin (42 kDa)

WT *Piezo1*$^{-/-}$ WT *Piezo1*$^{-/-}$

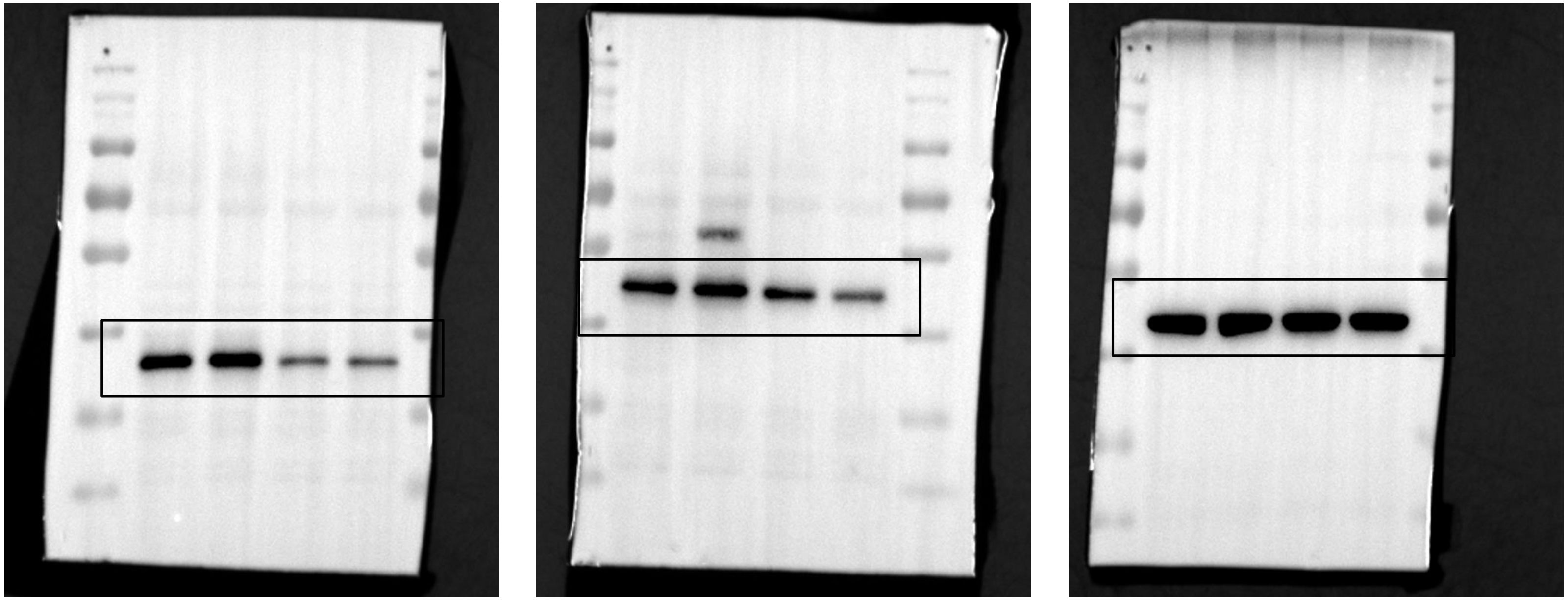

Fig.4C

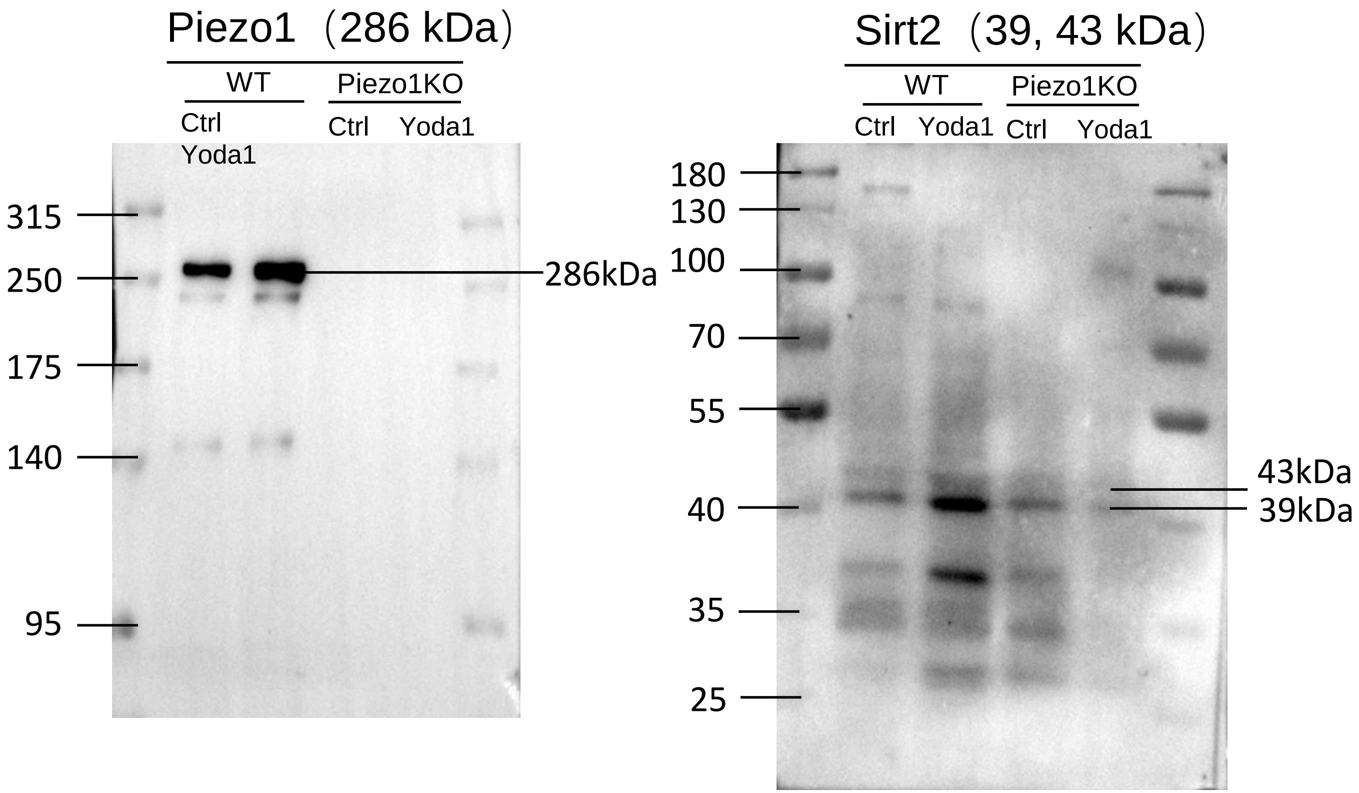

Piezo1 (286 kDa)
WT
Piezo1KO
Ctrl
Yoda1
Ctrl
Yoda1
315
250
175
140
95
286kDa
Sirt2 (39, 43 kDa)
WT
Piezo1KO
Ctrl
Yoda1
Ctrl
Yoda1
180
130
100
70
55
40
35
25
43kDa
39kDa

Fig.4C

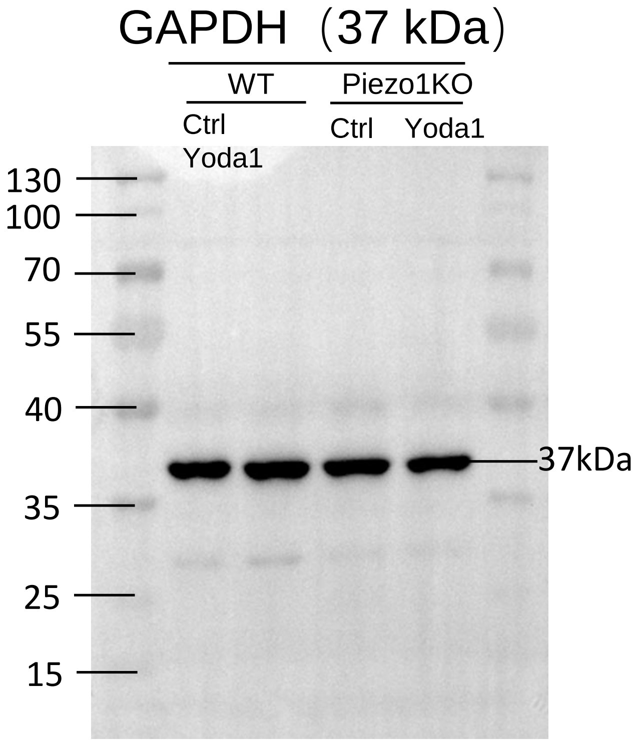
GAPDH (37 kDa)
WT
Piezo1KO
Ctrl
Yoda1
Ctrl
Yoda1
130
100
70
55
40
35
25
15
37kDa

Fig.4G

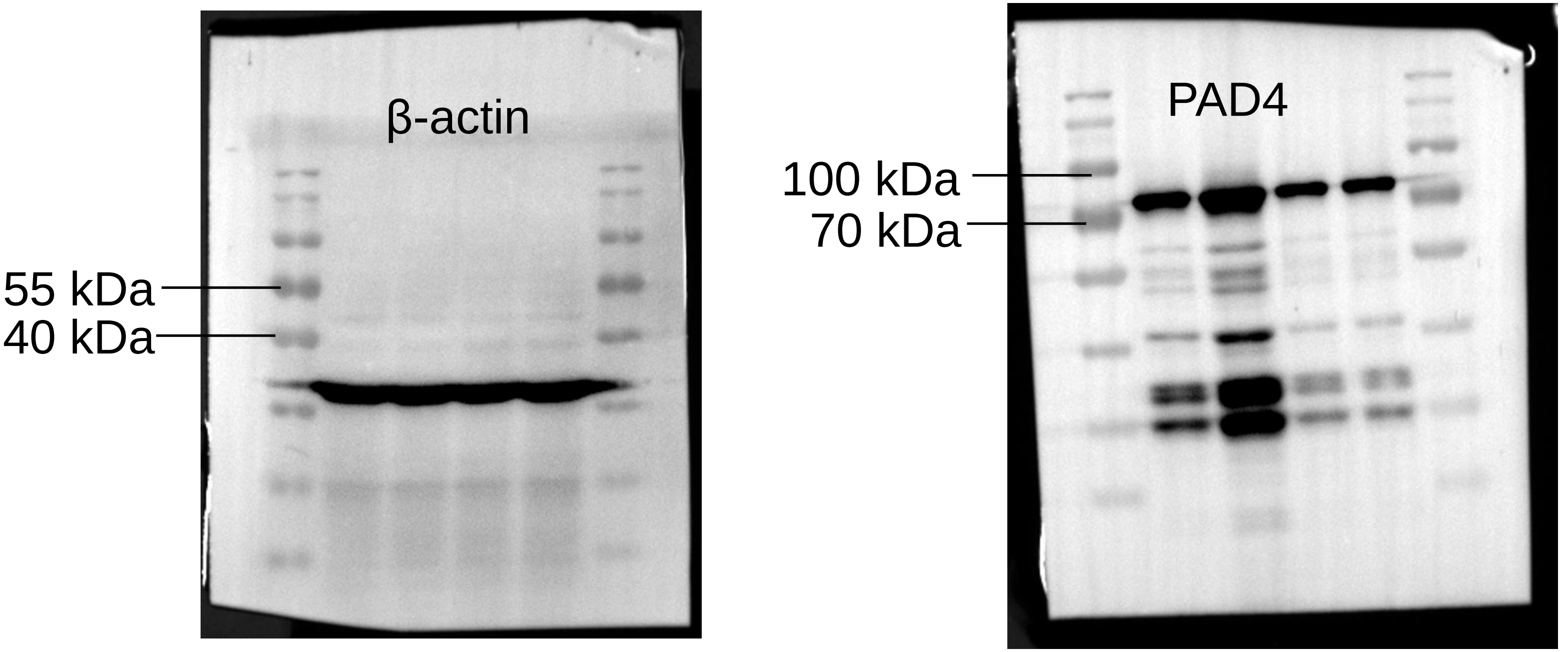

Fig.5G

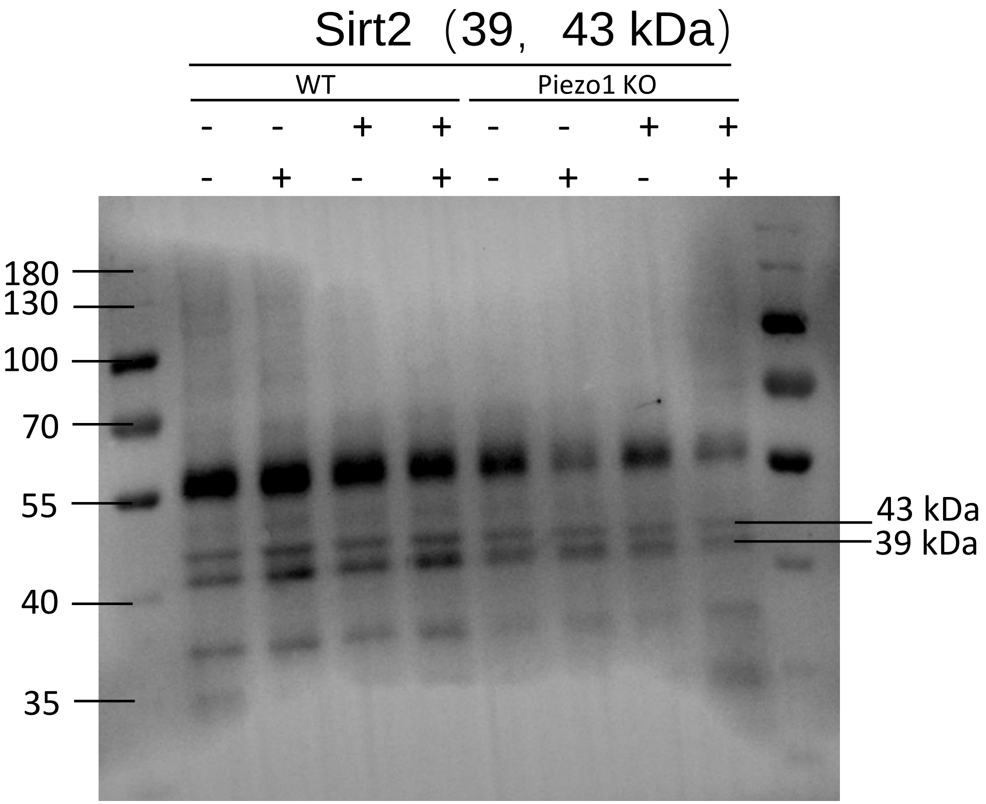

Fig.5G

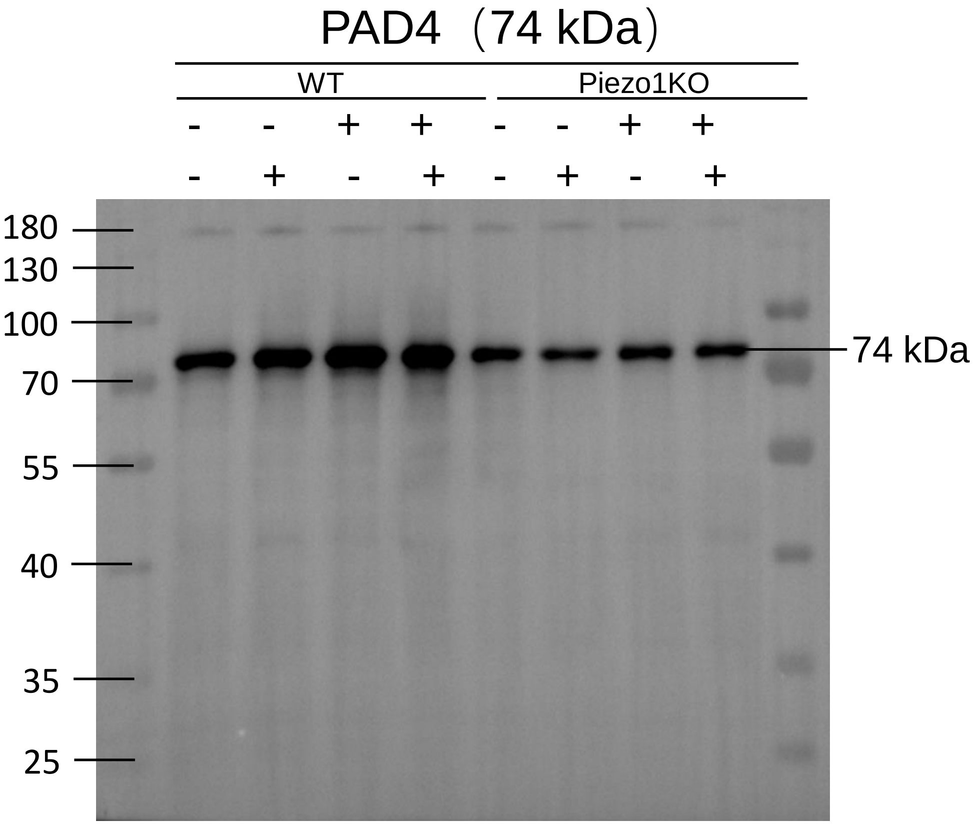

Fig.5G

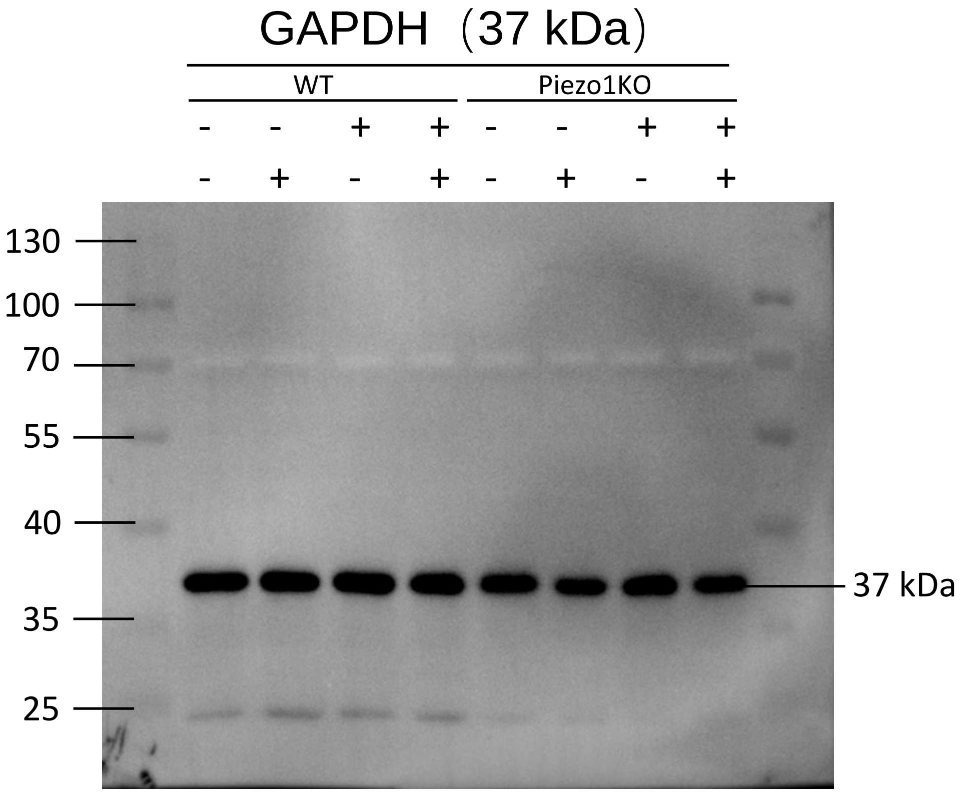

Fig.7C

Fig.7C

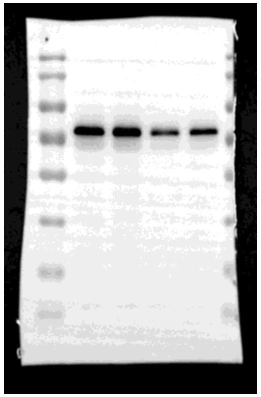

PAD4

Fig.7C

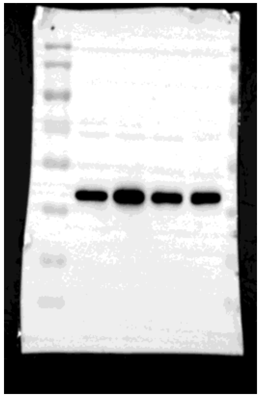

β-actin

Fig.7C

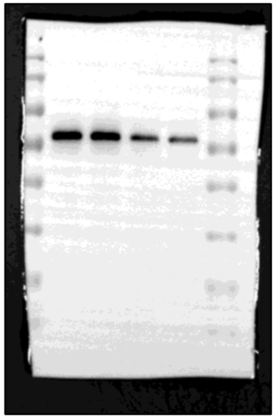

PAD4

Fig.7C

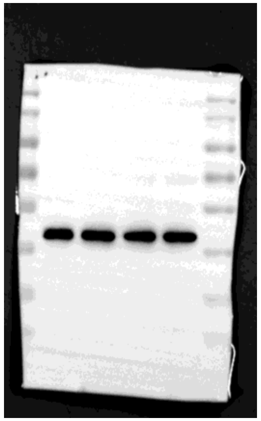

β-actin

Fig.S2E

β-Actin

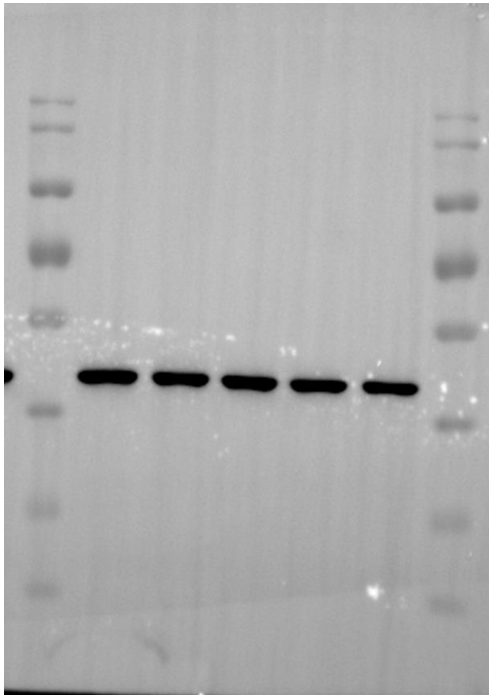

PAD4

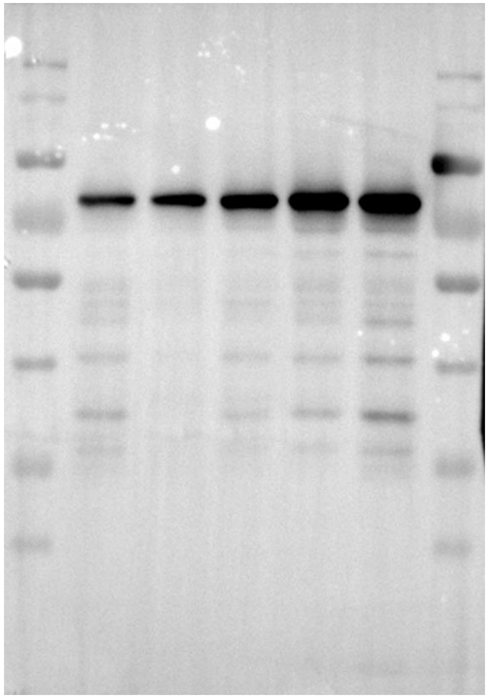

Fig.S4C

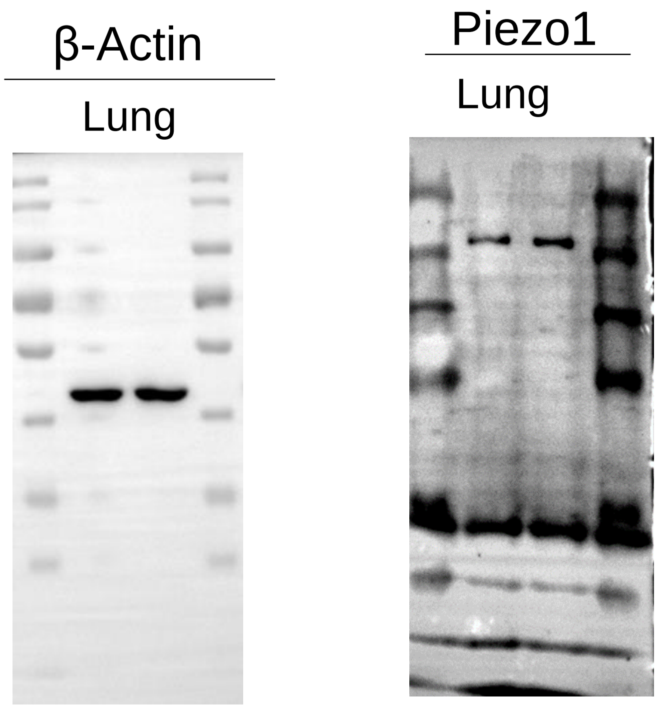

Fig.S6C

Fig.S6C

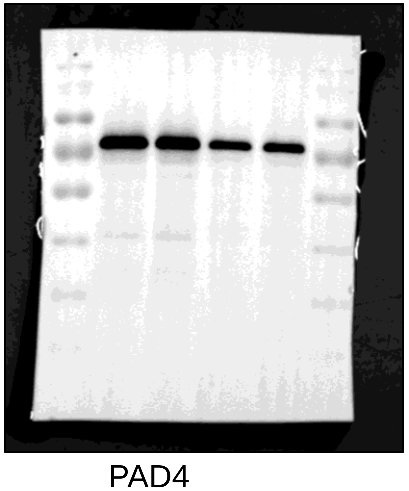

PAD4

Fig.S6C

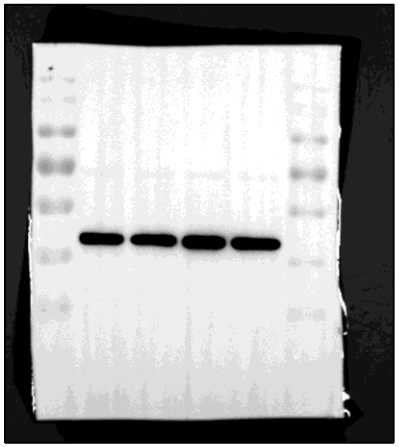

β-actin

Fig.S11A

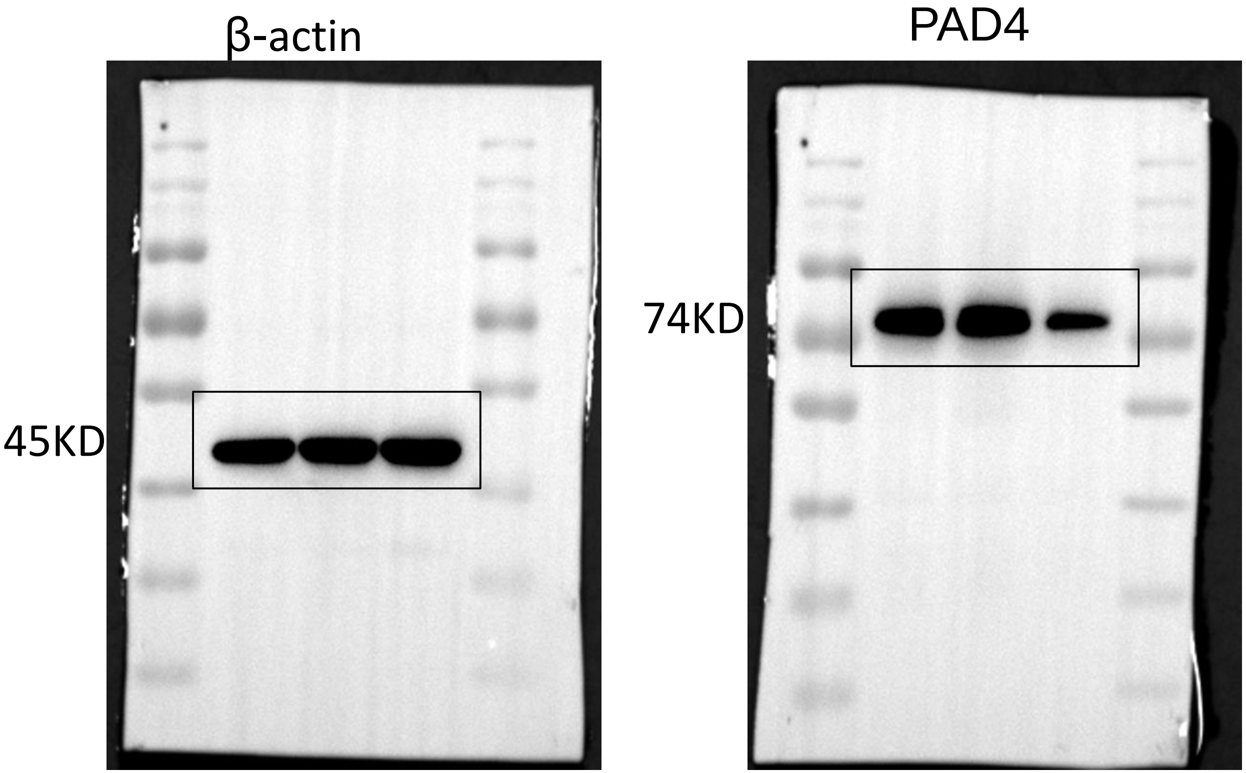

Fig.S14C

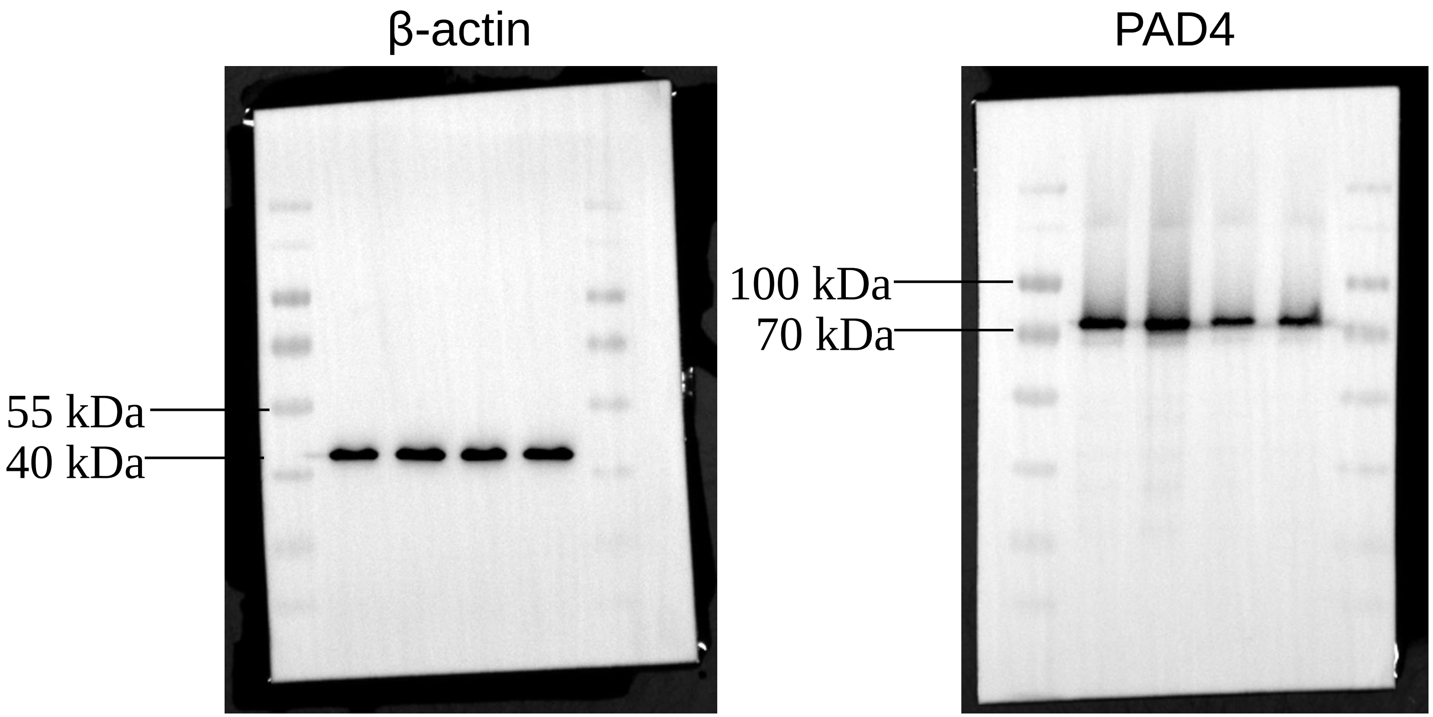

Supplement: Supplementary file 2 — Original Data [file 41419_2025_7395_MOESM2_ESM.pdf]
